# Innate Lymphoid Cells Are Required to Induce Airway Hyperreactivity in a Murine Neutrophilic Asthma Model

**DOI:** 10.3389/fimmu.2022.849155

**Published:** 2022-03-15

**Authors:** Anne-Charlotte Jonckheere, Sven F. Seys, Brecht Steelant, Tatjana Decaesteker, Kaat Dekoster, Jonathan Cremer, Ellen Dilissen, Dominique Schols, Yoichiro Iwakura, Greetje Vande Velde, Christine Breynaert, Rik Schrijvers, Jeroen Vanoirbeek, Jan L. Ceuppens, Lieven J. Dupont, Dominique M. A. Bullens

**Affiliations:** ^1^ Department of Microbiology, Immunology and Transplantation, Allergy and Clinical Immunology Research Group, KU Leuven, Leuven, Belgium; ^2^ Department of Chronic Diseases, Metabolism and Ageing, Laboratory of Respiratory Diseases and Thoracic Surgery, KU Leuven, Leuven, Belgium; ^3^ Department of Imaging and Pathology, Biomedical MRI Unit/Molecular Small Animal Imaging Center (MoSAIC), KU Leuven, Leuven, Belgium; ^4^ Department of Microbiology, Immunology and Transplantation, Laboratory of Virology and Chemotherapy, KU Leuven, Leuven, Belgium; ^5^ Centre for Animal Disease Models, Research Institute for Biomedical Sciences, Tokyo University of Science, Chiba, Japan; ^6^ Department of Public Health and Primary Care, Centre for Environment and Health, KU Leuven, Leuven, Belgium; ^7^ Clinical Division of Respiratory Medicine, UZ Leuven, Leuven, Belgium; ^8^ Clinical Division of Paediatrics, UZ Leuven, Leuven, Belgium

**Keywords:** innate lymphoid cells (ILCs), non-allergic asthma, murine model, neutrophilic inflammation, airway hyperreactivity

## Abstract

**Rationale:**

Non-allergic asthma is driven by multiple endotypes of which neutrophilic and pauci-granulocytic asthma have been best established. However, it is still puzzling what drives inflammation and airway hyperreactivity (AHR) in these patients and how it can be treated effectively. Recently, a potential role of the innate immune system and especially the innate lymphoid cells (ILC) has been proposed.

**Objective:**

In this study, we investigated the effects of LPS inhalation on airway inflammation and AHR as a potential model for elucidating the pathogenesis of non-allergic asthma.

**Methods:**

Wild-type (BALB/c), SCID, IL-17A^-/-^, and Rag2^-/-^ γC^-/-^ mice were endonasally exposed to lipopolysaccharide (LPS, 2 µg) on four consecutive days. Twenty-four hours after the last exposure, AHR to methacholine was assessed. Cytokine levels and ILC subpopulations were determined in lung tissue. Cellular differential analysis was performed in BAL fluid.

**Main Results:**

In this study, we developed a murine model for non-allergic neutrophilic asthma. We found that repeated endonasal applications of low-dose LPS in BALB/c mice led to AHR, BAL neutrophilia, and a significant increase in lung ILC3 as well as a significant increase in lung chemokines KC and MIP-2 and cytokines IL-1β, IL-17A, IL-22, and TNF. The adoptive transfer of ILC in Rag2^-/-^ γC^-/-^ mice showed that ILC played a causal role in the induction of AHR in this model. Antagonising IL-1β, but not IL-17A or neutrophils, resulted in a partial reduction in LPS-induced AHR.

**Conclusion:**

In conclusion, we report here a murine model for neutrophilic asthma where ILC are required to induce airway hyperreactivity.

## Introduction

Asthma affects 5% to 15% of the population of all ages (1). It is characterized by chronic airway inflammation and airway hyperreactivity (AHR) and has many phenotypes depending on the age of onset, allergic status, and severity of the disease, with different underlying types of inflammation (1, 2). Allergic eosinophilic asthma has been subject of extensive research, and many of the recent treatment strategies are based on experimental research findings in mice as several murine models, such as a house dust mite or ovalbumin model, already exist (2, 3). Allergic eosinophilic asthma, mostly early-onset asthma, can also be subdivided into early-onset mild allergic asthma or early-onset moderate to severe allergic asthma (1, 2), showing the heterogeneity of asthma. Both phenotypes are characterized with underlying eosinophilic inflammation, but the severity of the disease differs defined by the GINA guidelines as the level of treatment required to control asthma symptoms and exacerbations (4). Non-allergic asthma subtypes, often late-onset (5), which can be characterized by underlying eosinophilic, neutrophilic, or pauci-granulocytic inflammation, have been, however, less studied mechanistically. Still, there are data suggesting that their pathogenesis can be related to chronic exposure to pollutants, endotoxins, or viruses, to obesity and/or to extensive exercise (2). The activation of the airway epithelium by several stimuli can result in a cascade of cytokine releases followed by a chronic inflammatory reaction in the airways and possibly activation of neurogenic responses (6–8). The cornerstone of asthma treatment are the inhaled corticosteroids as controller treatment (4). Depending on the severity of asthma, different treatment options can be used as described in the GINA guidelines (4). However, some patients develop corticosteroid resistance (9), making it an urgent matter to fully understand the pathogenesis of allergic asthma, but more specifically of non-allergic asthma, as new therapies can then be found to obtain asthma control in patients with corticosteroid resistance.

Innate lymphoid cells (ILC) have surfaced as cells that could play an important role in the pathophysiology of asthma as a primary source of pro-inflammatory cytokines (10, 11). Three subtypes of ILCs have been identified, group 1 ILCs (ILC1), group 2 ILCs (ILC2), and group 3 ILCs (ILC3), each activated by different cytokines and each producing different effector cytokines (10). Recent studies have shown that ILC2s can contribute to induction of eosinophilic inflammation and AHR in murine allergic asthma models and that their activity is enhanced in asthmatic patients (11). On the other hand, ILC3-associated genes were increased in nasal brushings of adult-onset severe asthmatic patients, suggesting that these genes could be important in the pathogenesis of severe asthma with underlying neutrophilic inflammation (12).

The lack of mechanistic insight on non-allergic asthma is partly due to the lack of representative murine models exhibiting the typical features of AHR together with for example neutrophilic or pauci-granulocytic inflammation (1, 13). Therefore, we aimed to develop a murine model for non-allergic asthma with underlying neutrophilic inflammation and AHR in the absence of allergen sensitisation and exposure. Exposure to endotoxins (lipopolysaccharide (LPS)) was hypothesized to protect against the development of allergy and asthma in the so-called ‘Hygiene hypothesis’ (14–16). Mechanistically, LPS in combination with allergens was shown to inhibit type 2 inflammation in the airways, further endorsing the Hygiene hypothesis (17, 18). Contrastingly, endotoxins are also well-known triggers of asthma exacerbations in both allergic and non-allergic asthmatic patients (19). Exposure to LPS can shift allergic eosinophilic asthma toward a neutrophilic phenotype characterized by T-helper (Th)1 and Th17 responses (20, 21). Starkhammer and coworkers reported that small amounts of LPS could induce AHR and neutrophilic inflammation in a mouse model (22). We therefore studied the effects of repeated exposure to small doses of LPS on airway inflammation and AHR. We further hypothesized that cytokine production after exposure to LPS could then trigger ILC subsets and that these cells might fulfil an important mechanistic role in non-allergic asthma.

## Material and Methods

### Reagents

Lipopolysaccharide (LPS) from *Salmonella enterica* serotype Minnesota and fluticasone propionate (FP) were obtained from Sigma-Aldrich (St-Louis, MO). Anakinra (human interleukin-1 receptor antagonist, Kineret^®^) was kindly provided by Prof. Carine Wouters (UZ Leuven). Anti-Ly6G (clone 1A8) and rat IgG2 isotype (clone 2A3) were purchased from Bio X Cell (Lebanon, USA). Control liposomes and clodronate liposomes were obtained from Liposoma B.V. (The Netherlands). Pentobarbital (Dolethal^®^) from Vétoquinol SA (Aartselaar, Belgium) and isoflurane (Iso-Vet 1,000 mg/g) from Dechra Veterinary Products NV (Lille, Belgium) were obtained *via* the animal facility from KU Leuven.

### Animals

Eight- to 10-week-old male BALB/cOlaHsd mice purchased from Envigo (The Netherlands) were used to develop the neutrophilic asthma model. Eight- to 10-week-old male SCID (severe combined immune deficiency) mice and Rag2^-/-^ γC^-/-^ mice on BALB/c background (KU Leuven) were used to determine if ILCs play a role in the induction of airway hyperreactivity in our murine asthma model. The SCID mice have a spontaneous mutation in the *Prkdc* gene which leads to impairment of lymphoid differentiation and thus the deficiency of T and B cells. Interleukin (IL)-17A^-/-^ mice on BALB/c background were kindly provided by the Institute of Medical Science of the University of Tokyo, Japan. All mice were housed in specific pathogen-free conditions. Experiments were approved by the Ethical Committee for Animal Research KU Leuven (P025/2018) and comply with the ARRIVE guidelines.

### Neutrophilic Asthma Model

Wild-type, SCID, Rag2^-/-^ gC^-/-^, and IL-17A^-/-^ mice were endonasally challenged with 2 μg LPS (in a solution of 50 μl; 40 μg/ml) or 50 μl of saline (0.9% NaCl, B. Braun) on four consecutive days (**Supplementary Figure 1A**). All endonasal challenges were performed after anaesthesia with isoflurane. Twenty-four hours after the last challenge, *in vivo* lung scans were taken with µCT, lung function in the mice was assessed by invasive measurements, and an autopsy was performed obtaining blood, bronchoalveolar lavage, and lung tissue for ELISA, qPCR, single-cell suspension, and flow cytometry.

### Lung Function Measurements

Airway hyperreactivity was assessed 24 h after the last challenge with LPS or saline with the FlexiVent™ FX system (SCIREQ, Montreal, Canada), as previously described (23, 24). In brief, mice were anaesthetized by intraperitoneal injection of pentobarbital (120 mg/kg body weight) in a 1:33 dilution with saline (Dolethal^®^). Airway resistance (Rn) was measured at baseline and after nebulization of a 0-, 1.25-, 2.5-, 5-, 10-, and 20-mg/ml methacholine solution (Sigma-Aldrich) *via* the Quick Prime 3 perturbation (5 repeats with 15 s between each perturbation). Values with a COD > 0.9 are included in the analysis. Forced expiratory volume in 0.1 s (FEV_0.1_) was determined by using a negative pressure-driven forced expiratory (NPFE) volume perturbation with the help of the NPFE extension unit for mice (SCIREQ, Montreal, Canada). Detailed information about the measurement of FEV_0.1_ can be found in Devos et al. (23).

### Micro-Computed Tomography

Twenty-four hours after the last LPS challenge, micro-computed tomography (µCT) was performed to obtain *in vivo* lung scans of free-breathing mice (SkyScan 1278, Bruker µCT, Kontich, Belgium). Therefore, the mice were anesthetized with 1.5%–2% isoflurane in 100% oxygen and scanned according to a previously validated scan protocol (25). µCT scans were analysed with the software provided by the manufacturer (TSort, NRecon, DataViewer, and CTan) to respectively gate, reconstruct, visualize, and process µCT data as described previously (25–27).

### Neutrophil Depletion

To deplete the neutrophils, 250 µg of anti-Ly6G mAb or rat IgG2 isotype control was given intraperitoneally 24 h before the first challenge with LPS and on days 2 and 4 as shown in **Supplementary Figure 1B**. Twenty-four hours after the last LPS challenge, autopsy and lung function measurements were performed as described above.

### Neutralisation of the Cytokine IL-1β

To block the IL-1 receptor and thus the function of IL-1β, anakinra (50 µl of a 20-mg/ml solution) or sham (50 µl saline) was given endonasally 24 h before the first challenge of LPS together with the LPS challenge on four consecutive days in SCID mice (**Supplementary Figure 1C**). Lung function (FlexiVent) and autopsy were performed 24 h after the last LPS challenge, as described above.

### Glucocorticosteroid Treatment

To test the effect of glucocorticosteroids in this neutrophilic asthma model, 0.05 mg/kg FP or sham (50 µl saline + 10% dimethylsulfoxide (DMSO)) was given endonasally on days 3, 4, and 5, 1 hour after the challenge with LPS (**Supplementary Figure 1D**). The lung function in this part of the study was then assessed 48 h after the last challenge with LPS.

### Bronchoalveolar Lavage Fluid

Bronchoalveolar lavage (BAL) fluid was obtained by rinsing the lung three times with 750 µl of sterile saline (0.9% NaCl, B. Braun). BAL fluid was centrifuged for 10 min at 1,000 g at 4°C. Total viable cell count was determined *via* a Bruker hemocytometer using Trypan Blue staining (BioWhittaker^®^ Lonza). Cytospins in duplicate were made and stained with the DiffQuik method (Medical Diagnostics, Germany) for differential cell count. A total of 200 cells were counted to determine the percentage of macrophages, lymphocytes, neutrophils, and eosinophils in BAL fluid.

### Tight Junction mRNA Expression

Total RNA was isolated from lung tissue with the Qiagen Mini RNeasy Kit (Qiagen, Germantown, MD), as previously described (28–30). mRNA was reverse transcribed into cDNA using the high-capacity cDNA reverse transcription kit (Applied Biosystems™) according to the manufacturer’s instructions. Real-time qPCR was performed for mRNA of occludin, zonula occludens (ZO)-1, claudin-3, claudin-4, and claudin-18 (Bio-Rad, Hercules, CA, USA). Data were normalized to the geometric mean of the reference genes β-actin and β-2-microglobulin. Primer and probe sequences are found in **Supplementary Table 1**. cDNA plasmid standards were used to quantify the amount of target gene transcripts in unknown samples (31).

### Immunofluorescence Staining of Tight Junctions in the Epithelium, Haematoxylin–Eosin Staining, and Alcian Blue and Periodic-Acid Schiff Staining

Lung tissue was imbedded in paraffin, and 5-µm paraffin sections were made using a Microtome. Before overnight incubation at 4°C with antibodies against occludin (rabbit, anti-occludin, #71-1500, Thermo Fisher, Waltham, MA, USA), claudin 3 (rabbit, anti-claudin 3, #34-1700, Thermo Fisher), and claudin 4 (mouse, anti-claudin 4, #32-9400, Thermo Fisher), tissue sections were deparaffinized and antigen retrieval was performed. After overnight incubation, the sections were washed and either incubated with donkey anti-mouse antibody labelled with Alexa Fluor 488 (1/2000, #A21202, Thermo Fisher) or donkey anti-rabbit antibody labelled with Alexa Fluor 594 (1/2000, #A212007, Thermo Fisher) for 1 h at room temperature in the dark. Afterward, nuclei were counterstained with 4′,6-diamidino-2-phenylindole (DAPI) (Life Technologies, Grand Island, NY) and samples were mounted in FluorSave reagent (#345789-20, Merck, Darmstadt, Germany). Samples were analysed using a Zeiss LSM780 confocal microscope (Zeiss, Jena, Germany). Magnification used was ×25. Haematoxylin–eosin staining was done on paraffin sections from lung tissue as described in protocol by Fischer et al. (32). Lung paraffin coupes were deparaffinized and rehydrated before staining for 30 min with Alcian blue reagents (pH 2.5, Sigma-Aldrich). After rinsing with 3% acetic acid and tap water, tissue sections were oxidized in periodic acid for 10–30 min and immersed in Schiff’s reagent for 20 min. After washing for 10 min in running tap water, the sections were dehydrated and mounted (33). Images were taken with the Axiovert 200 M inverted microscope (Zeiss, Jena, Germany) at a magnification of ×40.

### Single Cell Suspension From Lung Tissue

Lungs were perfused with a phosphate-buffered saline (PBS) solution. Afterward the left lung lobe was collected, minced, and incubated with digestion medium (RPMI medium with 1 mg/ml collagenase type 2) for 45 min at 37°C. Red blood cells were lysed with an ammonium–chloride–potassium (ACK) lysing solution. The total lung viable cell count was determined *via* a Bruker hemocytometer using Trypan Blue colouring (BioWhittaker^®^ Lonza).

### Flow Cytometry

To characterize the innate lymphoid cells (ILCs), four million lung cells were incubated with Fixable Viability Dye eFluor™ 780 (Thermo Fisher Scientific, Waltham, MA USA) and pre-incubated with anti-mouse CD16/CD32 (Fc block; BD Biosciences). Afterward, the cells were incubated with a FITC anti-mouse lineage cocktail (CD3e, CD49b, CD11b, CD94, CD5, TCRγδ, CD19, Ter-119, Gr-1 and CD45RB), an AF700 anti-mouse CD45, a Pe-Dazzle594 anti-mouse Ly6G, a PE-Cy7 anti-mouse CD90.2, a PE anti-mouse CD127, a BV421 anti-mouse KLRG-1, and a BV711 anti-mouse Nkp46, permeabilized with the eBioscience™ FoxP3/transcription factor staining buffer set (Thermo Fisher Scientific, Waltham, MA USA) according to the manufacturer’s protocol and lastly stained with APC anti-mouse RORγT to identify the different subtypes of ILCs and exclude the lung neutrophils. All antibodies are purchased from BD Biosciences, except anti-CD94 and anti-Ly6G [purchased from BioLegend (San Diego, CA)] and anti-RORγT [obtained from Thermo Fisher Scientific (Waltham MA, USA)]. To identify the different dendritic cell subpopulations, 2 million lung cells were pre-incubated with anti-CD16/CD32 (Fc block, clone 2.4G2, BD Biosciences). Live/dead staining was done using the Zombie Aqua™ Fixable Viability Kit, and cells were incubated with a BV711 anti-mouse CD45 (clone 30-F11), a Pe-Dazzle 594 anti-mouse CD11c (clone N418), an APC-Cy7 anti-mouse MHCII (clone M5/114.15.2), a BV605 anti-mouse CD11b (clone M1/70), a BV421 anti-mouse CD103 (clone 2E7), an APC anti-mouse CD64 (clone X54-5/7.1), and a PE anti-mouse Siglec-H (clone 551) mAb all obtained from BioLegend (San Diego, CA). Data were acquired with a LSRFortessa SORP flow cytometer running DIVA software (BD Biosciences, Erembodegem, Belgium) and analysed with FlowJo 10.5.0 for Macintosh. The gating strategy for the ILC and DC panel is found in the supplementary data (**Supplementary Figures 2, 3**). More information about the used antibodies and the used markers (34, 35) is found in **Supplementary Table 2** and the configuration of the LSRFortessa SORP flow cytometer in **Supplementary Table 3**.

### ILC Transfer Experiment

The total lung ILC population was sorted from SCID mice with a BD FACS Aria IIu from BD Biosciences (Erembodegem, Belgium) *via* staining with anti-mouse fluorochrome-conjugated mAb against CD45, CD3e, CD49b, CD11b, CD94, CD5, TCRγδ, CD19, Ter-119 and CD45RB, Ly6-G, CD90.2, and CD127 (**Supplementary Table 2**). ILC cells (10^4^) were then intravenously injected in Rag2^-/-^ γC^-/-^ mice. Twenty-four hours after the adaptive transfer, mice were endonasally challenged with LPS (2 µg in 50 µl saline) on four consecutive days. Twenty-four hours after the last LPS challenge, lung function parameters were measured, and an autopsy was performed to obtain BAL fluid for differential cell count and lung tissue for ILC identification.

### Cytokine Levels in Lung Tissue Homogenates

One right lung lobe was homogenized with 500 µl PBS/BSA 5% with a Potter-Elvehjem homogenizer, and supernatant was collected after centrifugation at 10,000 g for 5 min. Lung granulocyte-macrophage colony-stimulating factor (GM-CSF), IL-1β, IL-33, IL-17A, keratinocyte-derived chemokine (KC), IL-6, IL-13, IL-4, IL-5, IL-22 IFN-γ, tumour necrosis factor (TNF)-⍺, and macrophage inflammatory protein (MIP)-2 concentrations were determined with the MSD U-Plex system according to the manufacturer’s instructions (Rockville, MD, USA). All cytokine levels were adjusted for lung weight (in mg). Detection limits were 0.54, 2.97, 0.35, 0.18, 0.22, 3.88, 12.9, 0.32, 0.37, 0.395, 0.23, 0.50, and 0.67 mg/ml, respectively.

### *In Vitro* Stimulation Experiments With Calu-3 Epithelial Cells

Calu-3 epithelial cells were seeded on 0.4 µm × 0.33 cm^2^ polyester Transwell inserts (Greiner Bio-One, Vilvoorde, Belgium) at a density of 100,000 cells per Transwell in Calu-3 culture medium consisting of Eagle minimal essential medium (Lonza) supplemented with 100 U/ml penicillin, 100 µg/ml streptomycin, 10% FCS, and 1% L-glutamine. The culture medium was refreshed every other day. After complete confluence, apical culture medium was removed, and cells were placed in an air–liquid interface (ALI). At day 21 in ALI, Calu-3 epithelial cells were used for stimulation experiments. Calu-3 epithelial cells were stimulated with 1 µg/ml LPS or medium for 6 or 48 h. After 6 h of stimulation, Calu-3 epithelial cells were collected, total RNA was isolated with the Qiagen Mini RNeasy Kit (Qiagen, Germantown, MD), and mRNA was reverse transcribed into cDNA using the high-capacity cDNA reverse transcription kit (Applied Biosystems™) according to the manufacturer’s instructions. Real-time qPCR was performed for mRNA of IL-1β, IL-6, IL-8, and IL-33 (Bio-Rad, USA). Data were normalized to the geometric mean of the reference genes β-actin and guanine nucleotide-binding protein subunit beta-2-like 1 (GNB2L1). Primer and probe sequences are found in **Supplementary Table 4**. cDNA plasmid standards were used to quantify the amount of target gene transcripts in unknown samples (31). Forty-eight hours after stimulation with LPS, cell culture supernatant was collected and IL-1β, IL-6, IL-8, and TNF-⍺ were measured by ELISA *via* an in-house protocol.

### Statistical Analysis

Statistical analysis was performed using GraphPad Prism v.7 for Macintosh (La Jolla, CA, USA). Normal distribution was studied for each group with a D’Agostino and Pearson normality test. One-way ANOVA with Tukey *post-hoc* test was used if the data were normally distributed and Kruskal–Wallis test with Dunn’s multiple-comparison test was used for non-parametric data. Two-way ANOVA with Bonferroni *post-hoc* test was used in the lung function analysis. The Mann–Whitney test was used to compare two groups with non-parametric data. A difference was considered significant when p < 0.05.

## Results

### Characterization of a Murine Model of Neutrophilic Airway Inflammation and Hyperreactivity

**Figure 1:**
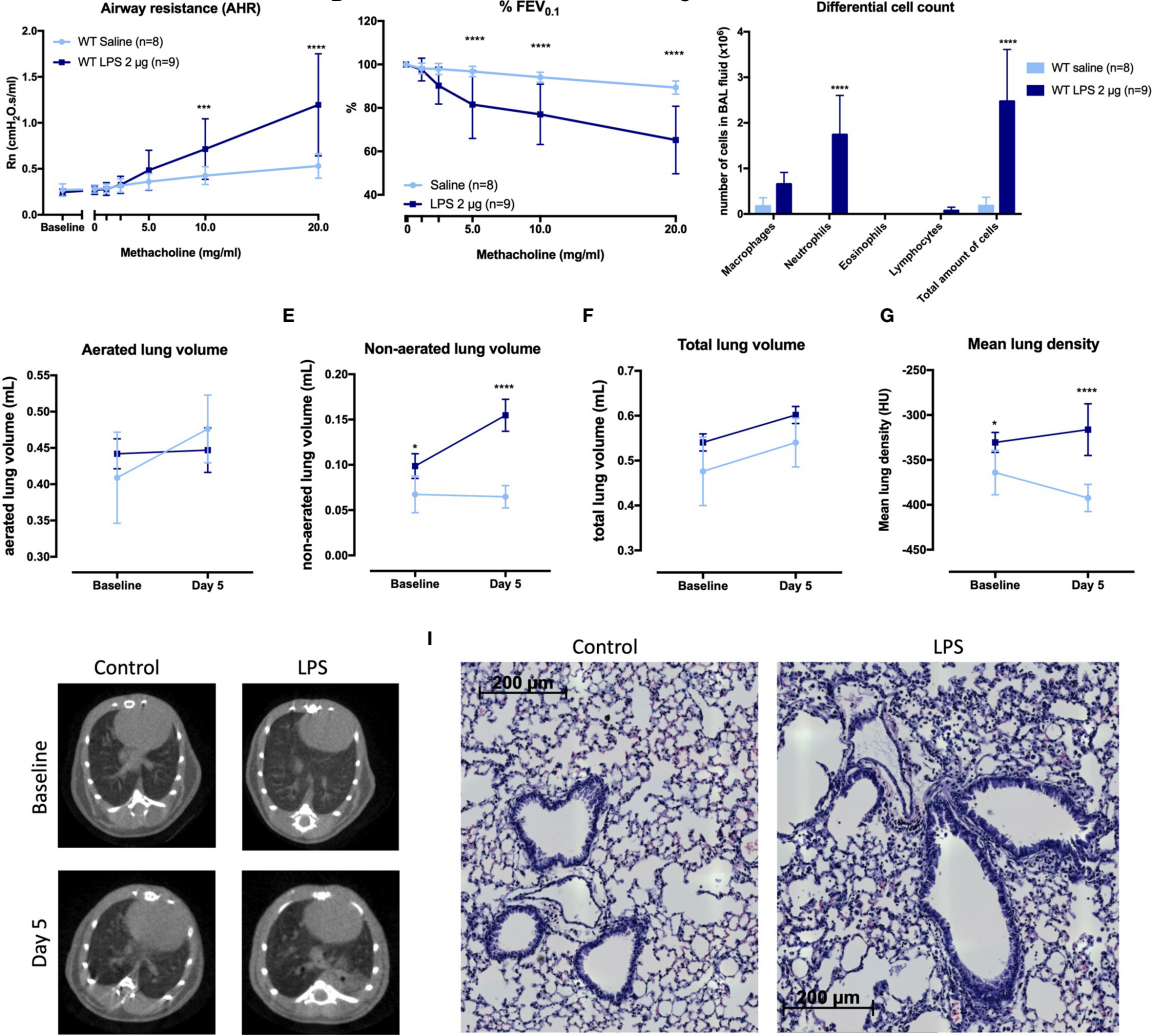
Characterization of a murine model for neutrophilic asthma. BALB/c wild-type (WT) mice were challenged with LPS (2 µg in 50 µl saline) *via* the nose on four consecutive days (**Supplementary Figure 1A**). Twenty-four hours after the last challenge with LPS, lung function parameters were measured with the FlexiVent and µCT was performed. Airway resistance [Rn, **(A)**] and FEV_0.1_ [%, **(B)**] before and after methacholine provocation (0–20 mg/ml) were measured in saline-treated mice (n = 8) and LPS-treated mice (n = 9) and a differential cell count **(C)** was performed on BAL fluid. Results from two independent experiments were combined. µCT-derived biomarkers were measured in saline-treated mice (light blue; n = 5) and LPS-treated mice (dark blue; n = 5): the aerated lung volume **(D)**, non-aerated lung volume **(E)**, total lung volume **(F)**, and mean lung density **(G)**. Representative µCT images at baseline (before saline or LPS challenges) and at day 5 are shown in **(H)** for control (saline-treated) mice and LPS-treated mice. Representative images of hematoxylin and eosin staining of lung tissue of saline-exposed and LPS-exposed mice at day 5 are shown in **(I)** (magnification ×40). Data are represented as mean with standard deviation. Two-way ANOVA was used to compare groups. *p < 0.05; ***p < 0.001; ****p < 0.0001. HU, Hounsfield unit; LPS, lipopolysaccharide.

We designed a murine model by 4 times daily application of 2 µg LPS endonasally (**Supplementary Figures 1, 4**). LPS-exposed mice had AHR as evidence by increased airway resistance (Rn) after methacholine inhalation, in combination with significantly lower forced expiratory volume in 0.1 second (%FEV_0.1_) compared to saline-exposed BALB/c mice (**Figures 1A, B**). Along with AHR, there was a significant increase in neutrophils in bronchoalveolar lavage (BAL) fluid of LPS-treated mice (**Figure 1C**).

To further evaluate the LPS effects on the airways, aerated and non-aerated lung volumes before (baseline) and after the last LPS challenge (day 5) were measured using micro-computed tomography (µCT). No differences were found in the aerated lung volume and the total lung volume at baseline (before any exposure to saline or LPS) or at day 5 between the two groups (**Figures 1D, F**). The non-aerated lung volume and the mean lung density (**Figures 1E, G**) were significantly increased at day 5 in LPS-treated mice compared to saline-treated mice, confirming lung inflammation. Representative images are shown in **Figure 1H**. Haematoxylin–eosin staining analysed by a blinded observer showed no clear lung structural differences between saline- and LPS-treated mice (**Figure 1I**). Mucus production determined *via* Alcian blue and periodic acid–Schiff (PAS) staining was not altered in LPS-treated mice, while Muc5ac mRNA expression was significantly decreased in LPS-treated mice (**Supplementary Figure 5**).

Furthermore, neutrophil-attracting chemokines keratinocyte-derived chemokine (KC) and macrophage inflammatory protein (MIP)-2 as well as the cytokines interleukin (IL)-1β, IL-17A, tumour necrosis factor (TNF)-⍺, IL-22, IL-6, IL-13, and interferon (IFN)-γ (**Table 1**) were significantly increased in the lungs of LPS-treated mice compared to control mice. Lastly, to fully characterize this model, we investigated whether the epithelial barrier was altered by LPS exposure (36). Lung tight-junction mRNA expressions for occludin, zonula occludens 1, claudin-3, claudin-4, and claudin-18 were indeed decreased in LPS-exposed mice compared to saline-exposed mice which was confirmed at the protein level for occludin, claudin-3, and claudin-4 (**Supplementary Figure 6**), indicative of a decrease in epithelial barrier integrity.

**Table 1:** Lung tissue cytokines in wild-type BALB/c mice exposed to saline or LPS.

	Saline-exposed mice (n = 8)	LPS-exposed mice (n = 8)	p-value
**IL-1β**	0.64 (0.49 – 1.53)	51.6 (11.5 – 90.7)	<0.0001
**IL-6**	0 (0 – 0.02)	7.8 (1.75 – 20.9)	0.0001
**TNF-⍺**	0.07 (0.05 – 0.12)	2.52 (0.49 – 6.36)	<0.0001
**IL-33**	55 (10 – 72.3)	3915 (57.5 – 9990)	0.258
**IFN-γ**	0 (0 – 0)	53 (16 – 173)	<0.0001
**IL-13**	0 (0 – 0)	0.81 (0.36 – 1.08)	<0.0001
**IL-17A**	1.5 (1.1 – 5.1)	77 (12 – 107)	0.0079
**IL-22**	1.17 (0 – 5.2)	16.8 (4.57 – 88.28)	0.0095
**KC**	1.12 (0.86 – 1.61)	16.57 (5.49 – 27.58)	0.0001
**MIP-2**	0.53 (0.44 – 1.42)	16 (2.5 – 33.6)	0.0001
**GM-CSF**	0.2 (0.19 – 0.4)	13 (7 – 17)	0.0002

Values are represented as pg per mg lung tissue and as median with 25 and 75 percentiles between brackets. The Mann – Whitney test was used to compare both groups.

### Effects of LPS Exposure on Innate Lymphoid Cells and Dendritic Cells

**Figure 2:**
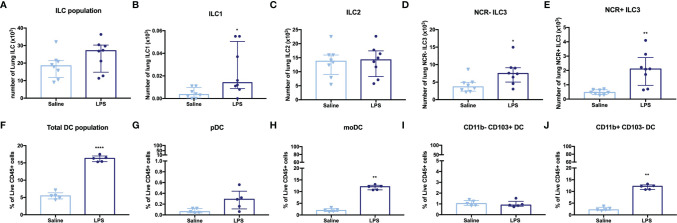
Lung innate lymphoid cells (ILC) and dendritic cells (DC) in LPS-challenged and control mice. BALB/c mice were endonasally challenged with LPS (2 µg in 50 µl saline) or saline on four consecutive days (**Supplementary Figure 1A**). Twenty-four hours after the last challenge with LPS, lung tissue was resected for cell isolation and the different innate lymphoid cell (ILC) and dendritic cell (DC) subsets were identified by staining with mAbs and flow cytometric analysis. **(A)** ILC population (CD45^+^ Lin^-^ CD90.2^+^ CD127^+^ cells). **(B)** ILC1 (CD45^+^ Lin^-^ CD90.2^+^ CD127^+^ KLRG1^-^ RORγT^-^ NKp46^+^). **(C)** ILC2 (CD45^+^ Lin^-^ CD90.2^+^ CD127^+^ KLRG1^+^ RORγT^-^ NKp46^-^). **(D)** NCR^-^ ILC3 (CD45^+^ Lin^-^ CD90.2^+^ CD127^+^ KLRG1^-^ RORγT^+^ NKp46^-^). **(E)** NCR^+^ ILC3 (CD45^+^ Lin^-^ CD90.2^+^ CD127^+^ KLRG1^-^ RORγT^+^ NKp46^+^). **(F)** DC population (CD45^+^ CD11c^+^ MHCII^+^). **(G)** pDC (CD45^+^ CD11c^+^ MHCII^+^ Siglec H^+^ CD11b^-^). **(H)** moDC (CD45^+^ CD11c^+^ MHCII^+^ CD64^+^). **(I)** CD11b^-^ CD103^+^ DC (CD45^+^ CD11c^+^ MHCII^+^ CD11b^-^ CD103^+^). **(J)** CD11b^+^ CD103^-^ DC (CD45^+^ CD11c^+^ MHCII^+^ CD11b^+^ CD103^-^). ILC (total and subpopulations) are shown in absolute numbers of cells recovered from one left lung lobe in saline-treated mice (n = 8) and LPS-treated (n = 8). The total DC and subpopulations are expressed as % of live CD45+ cells in saline-treated mice (n = 5) and LPS-treated mice (n = 5). All data are represented as individual values and median with interquartile range. The Mann–Whitney test was used to calculate the p-values. *p < 0.05; **p < 0.01; ****p < 0.0001.

To investigate the effects of LPS exposure on innate immunity, we identified the different ILC and DC subpopulations in lung tissue *via* flow cytometry. The total number of ILC in lung tissue (**Figure 2A**) of LPS-exposed mice was significantly increased, with a significant increase in ILC1, NCR^-^ ILC3, and NCR^+^ ILC3 numbers (**Figures 2B, D, E**), while the ILC2 subpopulation was not changed (**Figure 2C**). LPS exposure also induced significant changes in the DC subpopulations. We observed a significant increase in the total DC population (**Figure 2F**), which could be attributed to the monocytic-derived DC (moDC) and the CD11b^+^ CD103^-^ DCs (subpopulations linked to inflammatory environments, **Figures 2H, J**), in LPS-exposed mice compared to saline-exposed mice. No significant differences were found in pDC and CD11b^-^ CD103^+^ DC (**Figures 2G, I**).

### Effect of Glucocorticoid Treatment on Neutrophilic Inflammation and Airway Hyperreactivity

**Figure 3:**
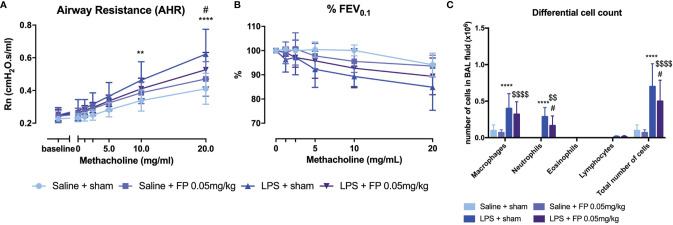
Effect of glucocorticoid treatment in the LPS-induced asthma model. BALB/c mice were exposed to LPS (2 µg in 50 µl saline) and fluticasone propionate (FP at 0.05 mg/kg in 50 µl) *via* the nose as shown in **Supplementary Figure 1D**. Forty-eight hours after the last challenge with LPS, lung function parameters were measured using the FlexiVent and BAL fluid obtained for differential cell count. Airway resistance [Rn, **(A)**] and FEV_0.1_ [%, **(B)**] before and after methacholine provocation (0–20 mg/ml) were measured in saline + sham-treated mice (n = 8), saline + FP-treated mice (n = 8), LPS + sham-treated mice (n = 8), and LPS + FP-treated mice (n = 8). **(C)** shows differential cell count in BAL fluid. Data are represented as mean with standard deviation, and two-way ANOVA was used to calculate the p-value. **p < 0.01; ****p < 0.0001 (saline + sham versus LPS + sham). ^#^p < 0.05; (LPS + sham versus LPS + FP). ^$$^p < 0.01; ^$$$$^p < 0.0001 (saline + FP versus LPS + FP).

Currently, the most common treatment for all asthma pheno- and endotypes is inhalation of glucocorticosteroids (4). However, patients with underlying neutrophilic inflammation might not have a similar beneficial effect of glucocorticoid treatment as patients with eosinophilic inflammation (37). Wild-type BALB/c mice were treated with fluticasone propionate (FP) after induction of neutrophilic inflammation (**Supplementary Figure 1D**). A partial but significant beneficial effect on AHR, as shown by Rn after methacholine exposure, was observed in LPS + FP-treated mice compared to LPS-treated mice (**Figure 3A**). No significant differences were found in %FEV_0.1_ (**Figure 3B**). Neutrophilic inflammation as reflected in BAL fluid composition was also partially reduced after FP treatment (**Figure 3C**). Treatment with FP had no influence on relative lung ILC proportions (**Supplementary Figure 7**), or lung cytokine production measured 48 h after LPS exposure (**Supplementary Table 5**).

### Role of IL-17A and Neutrophils in Induction of Airway Hyperreactivity

**Figure 4:**
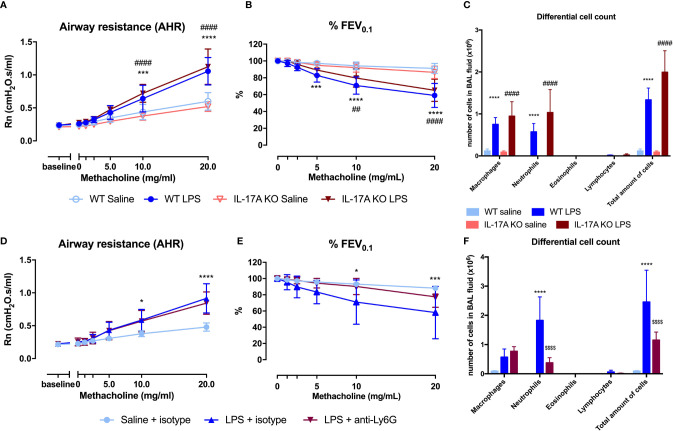
Analysis of the role of IL-17A and neutrophils in induction of airway hyperreactivity in the LPS model. IL-17A^-/-^ mice (on BALB/c background) were exposed to LPS (2 µg in 50 µl saline) *via* the nose for four consecutive days (**Supplementary Figure 1A**). Twenty-four hours after the last LPS challenge, lung function parameters were measured using the FlexiVent and BAL fluid was obtained for differential cell count. Airway resistance [Rn, **(A)**] and FEV_0.1_ [%, **(B)**] before and after methacholine provocation (0–20 mg/ml) were measured in saline-treated IL-17A^-/-^ mice (n = 8) and LPS-treated IL-17A^-/-^ mice (n = 8). **(C)** Differential cell count in BAL fluid of the same mice. **(D–F)** Neutrophils were depleted by anti-Ly6G mAb (250 μg injected intraperitoneally on days -1, 1, and 3 as shown in **Supplementary Figure 1B**). Twenty-four hours after the last LPS challenge, lung function parameters were measured using the FlexiVent and BAL fluid obtained for differential cell count. Airway resistance [Rn, **(D)**] and FEV_0.1_ [%, **(E)**] before and after methacholine provocation (0–20 mg/ml) were measured in saline-treated isotype injected wild type mice (n = 5), LPS-treated isotype injected wild-type mice (n = 5) and LPS-treated and anti-Ly6G-injected wild-type mice (n = 8). **(F)** Differential cell count in BAL fluid of the same mice. *p <0.05; ***p < 0.001; ****p < 0.0001 (WT saline versus WT LPS and saline + isotype versus LPS + isotype). ^##^p < 0.01; ^####^p < 0.0001 (IL17A^-/-^ saline versus IL-17A^-/-^ LPS). ^$$$$^p < 0.0001 (LPS + isotype versus LPS + anti-Ly6G).

As IL-17A has been linked to neutrophilic asthma and was significantly increased in lungs of LPS exposed mice (**Table 1**), we further investigated its contribution to the induction of AHR and airway inflammation. IL-17A^-/-^ mice (on BALB/c background) exposed to LPS (**Supplementary Figure 1A**) showed AHR in response to increasing doses of methacholine, with a significantly increased Rn (comparable to LPS exposed wild-type BALB/c) and significantly decreased %FEV_0.1_ compared to saline-treated IL-17A^-/-^ mice (**Figures 4A, B**). These data argue against an essential role for IL-17A in the induction of AHR in this model. Furthermore, neutrophilic inflammation was similarly present in LPS-exposed wild-type mice and in LPS-exposed IL-17A^-/-^ mice (**Figure 4C**). We confirmed that there was no IL-17A production in LPS-exposed IL-17A^-/-^ mice, but lung KC, MIP-2, IL-1β, and TNF-⍺ levels were all elevated in LPS-exposed IL-17A^-/-^ mice compared to saline-exposed IL-17A^-/-^ mice (**Supplementary Table 6**). The lung ILC subpopulations of LPS-exposed IL-17A^-/-^ mice were significantly altered with an increase of % NCR^+^ ILC3 together with a decrease in % ILC2 compared to saline-exposed IL-17A^-/-^ mice, which is similar to the relative ILC proportions seen in LPS-exposed wild-type mice (**Supplementary Figure 8**).

To assess the role of neutrophils in the induction of AHR, we depleted the neutrophils using anti-Ly6G mAb (**Supplementary Figure 1B**). Mice exposed to LPS showed an increase in Rn in response to increasing doses of methacholine independently of treatment with anti-Ly6G mAb, suggesting that neutrophils do not contribute to the induction of AHR in this model (**Figure 4D**). No change in %FEV_0.1_ was observed after treatment with anti-Ly6G mAb in LPS-exposed mice (**Figure 4E**). As expected, a significant reduction of neutrophils was noted in BAL fluid from mice exposed to LPS and treated with anti-Ly6G mAb when compared to LPS-exposed untreated mice (**Figure 4F**). On the contrary, lung KC, MIP-1, IL-1β, IL-17A, TNF-⍺, IL-6, and IL-13 levels were even higher after treatment with anti-Ly6G mAb than without neutrophil depletion (**Supplementary Table 7**). No significant differences in lung ILC proportions were found between LPS-exposed isotype-injected mice compared to LPS-exposed anti-Ly6G-injected mice (**Supplementary Figure 9**).

### Adaptive Immunity Is Not Required for LPS-Induced Airway Inflammation and Hyperreactivity

**Figure 5:**
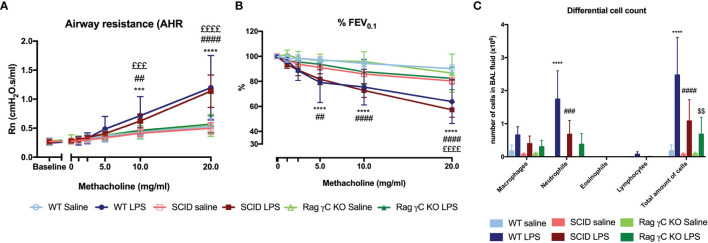
Comparison of the LPS asthma model in different mouse strains. Wild-type BALB/c mice (WT), SCID mice, and Rag2^-/-^ γC^-/-^ mice (on BALB/c background) were exposed to LPS (2 µg in 50 µl saline) *via* the nose on four consecutive days (**Supplementary Figure 1A**). On day 5, lung function parameters were measured using the FlexiVent and BAL fluid was obtained to determine the differential cell count. Airway resistance [Rn, **(A)**] and FEV_0.1_ [%, **(B)**] before and after methacholine provocation (0–20 mg/ml) were measured in saline-treated WT mice (n = 8), LPS-treated WT mice (n = 9), saline-treated SCID mice (n = 10), LPS-treated SCID mice (n = 10), saline-treated Rag2^-/-^ γC^-/-^ mice (n = 10), and LPS-treated Rag2^-/-^ γC^-/-^ mice (n = 10). Data are represented as mean with standard deviation. Differential cell count **(C)** was identified in BAL fluid of the same mice and represented as mean with standard deviation. ***p < 0.001; ****p < 0.0001 (WT saline versus WT LPS). ^##^p < 0.01; ^###^p < 0.001; ^####^p < 0.0001 (SCID saline versus SCID LPS). ^£££^p < 0.001; ^££££^p < 0.0001 (WT LPS versus Rag2^-/-^ γC^-/-^ LPS). ^$$^p < 0.01 (Rag2^-/-^ γC^-/-^ saline versus Rag2^-/-^ γC^-/-^ LPS).

To confirm that the adaptive immunity is not functional in this model, we used SCID mice. Both wild-type and SCID mice exposed to LPS were hyperreactive in response to increasing doses of methacholine as shown with the significant Rn increase and the significant decrease in %FEV_0.1_ when compared to saline-exposed wild-type or SCID mice respectively (**Figures 5A, B**). Neutrophilic inflammation was found in all mice exposed to LPS, with a significant increase in both wild-type mice and SCID mice, compared to the saline-exposed wild-type and SCID mice (**Figure 5C**). Also, KC, IL-1β, and IL-17A were significantly increased in lung tissue of SCID mice treated with LPS compared to saline-treated SCID mice (**Table 2**). In SCID mice treated with LPS, %NCR^-^ ILC3 were elevated compared to saline-treated SCID mice, together with a decrease in %ILC2 (similarly to the ILC proportions in wild-type mice; **Supplementary Figure 10**).

**Table 2:** Lung cytokine production in lung tissue of wild-type BALB/c mice, SCID mice, and Rag2^-/-^ γC^-/-^ mice nasally exposed to saline or LPS.

	WT mice			SCID mice			Rag2^-/-^ γC^-/-^ mice		
	Saline	LPS	p-value	Saline	LPS	p-value	Saline	LPS	p-value
**IL-1β**	0.33 (0.19 – 0.49)	48.6 (23.8 – 71.3)	0.0011	0.20 (0.16 – 0.28)	27.7 (18.5 – 44.5)	<0.0001	25.6 (20.6 – 36.6)	357 (156.7 – 629.6)	0.147
**IL-17A**	0.8 (0.5 – 1.2)	32.2 (15.9 – 93.9)	0.0002	0.9 (0.6 – 1.2)	6.2 (4.7 – 13.3)	0.0041	0.7 (0.4 – 0.9)	1.3 (0.7 – 1.7)	0.582
**KC**	22.9 (13.2 – 28.8)	54.9 (36.7 – 68.4)	0.0031	25.1 (23.3 – 33.6)	48.4 (36.3 – 72.5)	0.0054	26.3 (21.2 – 29.9)	28.5 (26 – 31.2)	0.717
**IFN-γ**	0.5 (0.3 – 0.7)	72.7 (50.9 – 129)	0.0044	0.2 (0.09 – 0.5)	55.7 (32.7 – 179.9)	<0.0001	0.4 (0.3 – 0.6)	0.7 (0.4 – 0.9)	>0.999
**GM-CSF**	1.5 (1.1 – 1.6)	10.2 (9.3 – 16.3)	0.0091	1.3 (1.01 – 1.5)	15.9 (11.5 – 27)	<0.0001	1.1 (0.97 – 1.4)	2.2 (1.7 – 2.9)	0.055
**IL-4**	0 (0 – 0)	1.5 (1.4 – 2.8)	0.0005	0 (0 – 0.2)	1.4 (0.97 – 1.9)	0.0011	0.5 (0.2 – 0.6)	0.9 (0.7 – 1.4)	0.292
**IL-5**	0 (0 – 0.7)	1.7 (0.3 – 2.3)	0.0534	0 (0 – 0.98)	1.6 (0.8 – 2.6)	0.0436	0 (0 – 0)	0 (0 – 0.6)	>0.999
**IL-13**	11 (0 – 13.7)	0 (0 – 6.2)	0.6444	0 (0 – 0)	11.5 (0 – 19.9)	0.0183	0 (0 – 10.6)	0 (0 – 12.3)	>0.999
**IL-33**	4176 (3613 – 5143)	12554 (8880 – 19341)	0.0039	3641 (3256 – 3897)	12761 (10435 – 14451)	<0.0001	4928 (4201 – 5919)	12723 (6958 – 17313)	0.156

Values are expressed as pg per mg lung tissue and presented as median with 25 and 75 percentiles between brackets. Kruskal – Wallis with Dunn’s post hoc test was used to calculate the p-value. LPS, lipopolysaccharide.

### Role of IL-1β in Induction of Airway Hyperreactivity

**Figure 6:**
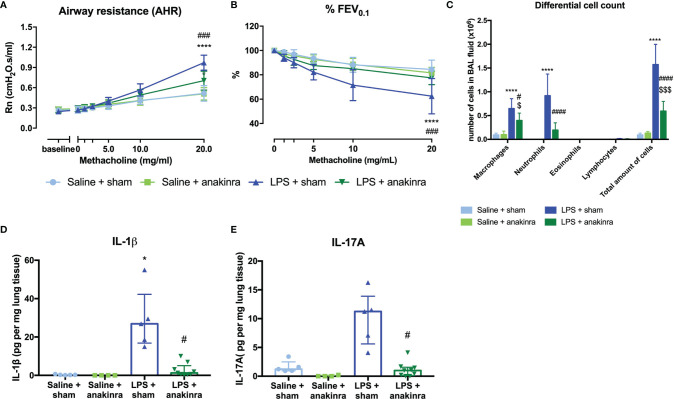
Effect of anakinra in the LPS model. SCID mice were endonasally treated with anakinra (50 μl of a 20 mg/ml solution) on days -1, 1, and 3 together with the four endonasal challenges with LPS (2 µg in 50 µl saline), as shown in **Supplementary Figure 1C**. Twenty-four hours after the last challenge with LPS, lung function parameters were measured using the FlexiVent, and BAL fluid was obtained for differential cell count and lung tissue for cytokine analysis. Airway resistance [Rn, **(A)**] and FEV_0.1_ [%, **(B)**] before and after methacholine provocation (0–20 mg/ml) were measured in saline + sham-treated mice (n = 7), saline + anakinra-treated mice (n = 7), LPS + sham-treated mice (n = 7) and LPS + anakinra-treated mice (n = 7). Differential cell count in BAL fluid is shown in **(C)**. Lung IL-1β and IL-17A levels corrected for lung weight are shown in **(D, E)** in the different groups. Data are represented as mean with standard deviation in **(A–C)** and as individual values with median and interquartile range in **(D, E)**. Two-way ANOVA was used in **(A–C)** and Kruskal–Wallis with Dunn’s *post hoc* test was used to calculate the p-values. *p < 0.05; ****p < 0.0001 (saline + sham versus LPS + sham). ^#^p < 0.05; ^###^p < 0.001; ^####^p < 0.0001 (LPS + sham versus LPS + anakinra). ^$^p < 0.05; ^$$$^p < 0.001 (saline + anakinra versus LPS + anakinra).

As IL-1β is increased in the lungs of LPS-exposed mice and has been linked to obesity-associated AHR (28, 38), we investigated whether IL-1β might be important to induce AHR in this model. Therefore, we treated saline- and LPS-exposed SCID mice with anakinra, an IL-1 receptor antagonist (**Supplementary Figure 1C**). Treatment with anakinra in LPS-exposed SCID mice indeed led to a decrease in AHR as evaluated by the response to increasing doses of methacholine, both shown in Rn and %FEV_0.1_, compared to LPS-exposed SCID mice without anakinra treatment (**Figures 6A, B**). Airway inflammation was also significantly decreased in LPS-exposed SCID mice treated with anakinra, with lower numbers of neutrophils in BAL fluid (**Figure 6C**). Both lung IL-1β and IL-17A were decreased in anakinra-treated LPS-exposed SCID mice compared to untreated LPS-exposed SCID mice (**Figures 6D, E**). Treatment with anakinra did not induce changes in lung ILC proportions of LPS exposed SCID mice (**Supplementary Figure 11**). Stimulation of Calu-3 epithelial cells with LPS (1 µg/ml) *in vitro* induced an increase in IL-1β on mRNA and a slight increase in protein level, making epithelial cells a possible source of IL-1β after triggering with LPS (**Supplementary Figure 12A, E**). In addition, IL-6, IL-8, and IL-33 mRNA levels were elevated after *in vitro* stimulation of Calu-3 cells with LPS together with increased protein levels of IL-6 and IL-8 as measured in cell culture supernatant (**Supplementary Figure 12**).

### Innate Lymphoid Cells Are Required for the Induction of Airway Hyperreactivity

Since ILCs contribute to allergic asthma (39), we investigated if ILCs play a role in AHR induction. Consequently, we first investigated if we could induce similar lung responses in Rag2^-/-^ γC^-/-^ mice (no adaptive immune system and no ILCs; **Supplementary Figure 13**) as in SCID mice (which do have ILC). Importantly, LPS-exposed Rag2^-/-^ γC^-/-^ mice were not hyperreactive (**Figures 5A, B**). LPS-exposed Rag2^-/-^ γC^-/-^ mice still showed an increase in lung IL-1β levels and in BAL neutrophils compared to saline-exposed Rag2^-/-^ γC^-/-^ mice (**Figure 5C** and **Table 2**).

**Figure 7:**
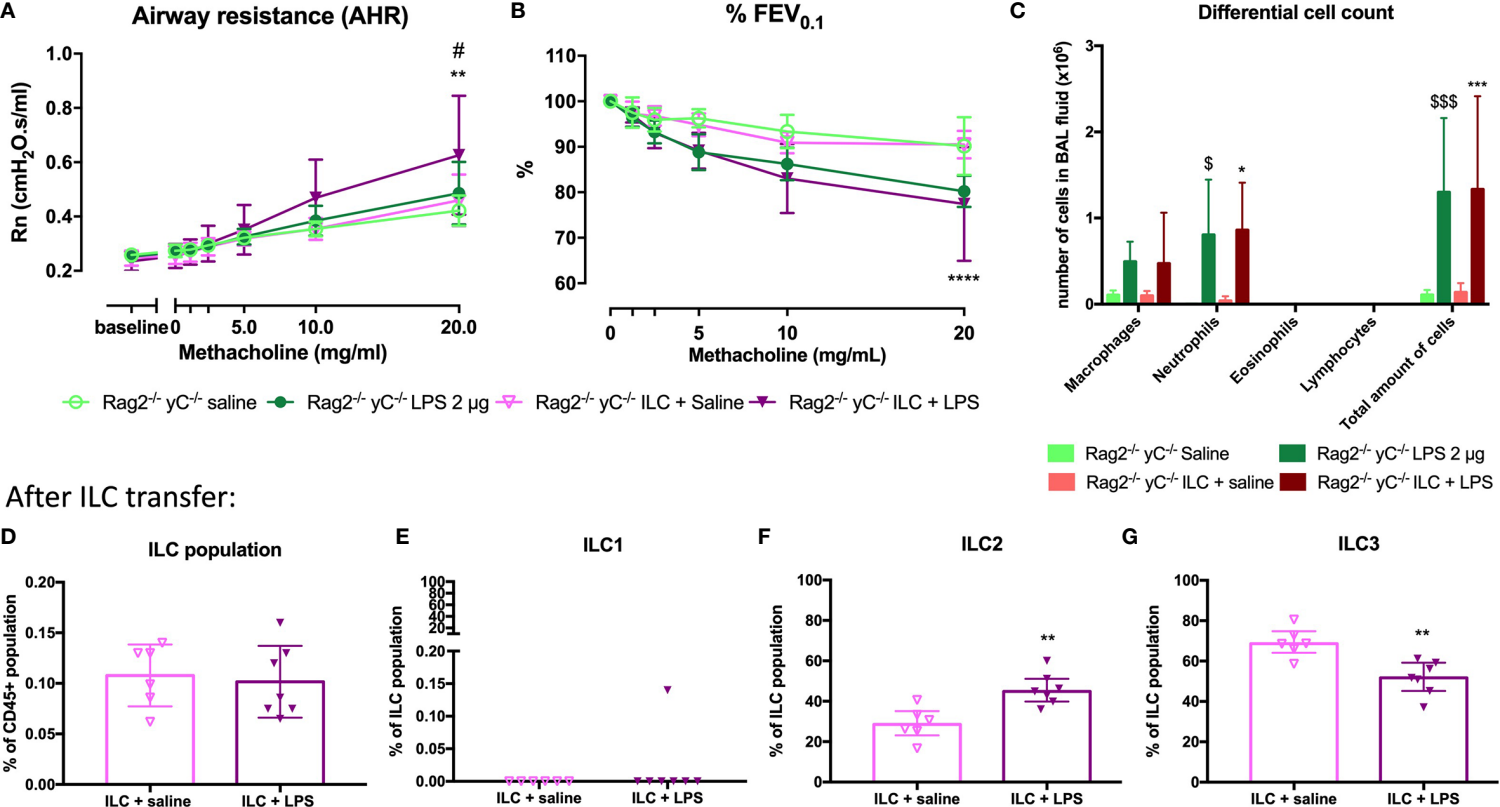
Effect of adoptive transfer of ILC in Rag2^-/-^ γC^-/-^ mice. ILC cells were isolated by sorting from lung tissue of SCID mice and intravenously injected (10^4^ cells) in Rag2^-/-^ γC^-/-^ mice. Twenty-four hours after the adoptive transfer, Rag2^-/-^ γC^-/-^ mice were endonasally exposed to LPS (2 µg in 50 µl saline) on four consecutive days. Twenty-four hours after the last LPS challenge, lung function parameters were measured using the FlexiVent, and BAL fluid was obtained for differential cell count and lung tissue for ILC identification. Airway resistance [Rn, **(A)**] and FEV_0.1_ [%, **(B)**] before and after methacholine provocation (0–20 mg/ml) were measured in saline-treated Rag2^-/-^ γC^-/-^ mice (n = 5), LPS-treated Rag2^-/-^ γC^-/-^ mice (n = 6), saline-treated Rag2^-/-^ γC^-/-^ mice after ILC transfer (n = 6), and LPS-treated Rag2^-/-^ γC^-/-^ mice after ILC transfer (n = 6). Data are represented as mean with standard deviation. Two-way ANOVA was used to determine significant differences. Differential cell count in BAL fluid of the same mice is shown in **(C)**. Total ILC population **(D)**, ILC1 **(E)**, ILC2 **(F)**, ILC3 **(G)** were identified in lung tissue *via* flow cytometry in saline-treated Rag2^-/-^ γC^-/-^ mice with ILC transfer (n = 6) and LPS-treated Rag2^-/-^ γC^-/-^ mice with ILC transfer (n = 6). These data are represented as individual values with median and interquartile range. Results from three independent experiments were combined. The unpaired t-test **(D, F, G)** or Mann–Whitney test **(E)** were used to calculate the p-value. *p < 0.05; **p < 0.01; ****p < 0.0001 (ILC + saline versus ILC + LPS). ^#^p < 0.05 (LPS versus ILC + LPS); ^$^p < 0.05; ^$$$^p < 0.001 (saline versus LPS).

To confirm that ILCs are required for the induction of AHR, the total ILC population was sorted from SCID mice, characterized (**Supplementary Figure 10**), and transferred to the lungs of Rag2^-/-^ γC^-/-^ mice *via* intravenous injection. After ILC transfer and LPS applications in Rag2^-/-^ γC^-/-^ mice, we were again able to demonstrate AHR in response to LPS (as evidenced by the significantly increased Rn in response to increasing doses of methacholine) and %FEV_0.1_ was significantly decreased, compared to saline-exposed Rag2^-/-^ γC^-/-^ mice without ILC transfer or Rag2^-/-^ γC^-/-^ mice transferred with ILC but saline-exposed (**Figures 7A, B**). Independent of the ILC transfer, neutrophils were significantly increased in Rag2^-/-^ γC^-/-^ mice treated with LPS which further supports our previous conclusion that neutrophils are not important for induction of AHR (**Figure 7C**). Subsequently, we characterized the lung ILC subpopulations of Rag2^-/-^ γC^-/-^ mice that received a total ILC transfer. We found a significantly higher proportion of lung ILC2 (%) and a lower ILC3 proportion in LPS-exposed Rag2^-/-^ γC^-/-^ mice that received ILCs compared to the saline-treated Rag2^-/-^ γC^-/-^ mice with an ILC transfer (**Figures 7F, G**). No changes in ILC1 and total ILC proportions were observed in LPS-exposed Rag2^-/-^ yC^-/-^ mice that received ILCs (**Figures 7D, E**).

## Discussion

In this study, a murine model resembling non-allergic neutrophilic asthma has been developed. By applying 2 µg LPS on four consecutive days in BALB/c mice, lung neutrophilic inflammation and AHR as evidenced by the response to methacholine were induced. LPS is often also used for murine models for acute lung injury also characterized by LPS-induced neutrophilic inflammation (40). However, the concentrations used in these models to induce acute lung injury are ten to fifty times more than used in this model (40–42) and airway hyperreactivity is often not included in these studies. Interestingly, Khadangi et al. showed that no changes in respiratory mechanisms could be observed in their model used for acute lung injury (42). By lowering the dose of LPS, we tried to mimic several features of non-allergic asthma with neutrophilic inflammation such as AHR and neutrophilic inflammation. Furthermore, the imaging technique µCT was utilized to identify inflamed regions in the lungs of LPS-instilled mice. Lung IL-1β, IL-17A, and TNF-α, all cytokines related with neutrophilic inflammation together with the neutrophil-attracting chemokines KC and MIP-2, were increased in LPS-instilled mice compared to control mice. We consider those features of relevance to endorse this model for neutrophilic asthma. Our results are in line with the study by Starkhammer et al., where LPS-instilled BALB/c mice also developed AHR and neutrophilic inflammation (22). In contrast with this study, we found an increase in lung cytokines IL-1β, IL-17A, and TNF-α together with chemokine KC in LPS-instilled mice. Several other experimental models with neutrophilic airway inflammation and AHR have been described, but all combined with an allergen such as ovalbumin or house dust mite (HDM), making them models for LPS-induced exacerbation or modulation of allergic asthma or mixed granulocytic asthma (43, 44). On the other hand, no clear increase in mucus production was noticed in our model which seems to be in contrast to the increased mucus production observed in asthmatic patients (45). As Muc5ac expression is often linked to type 2 inflammation (45) and this model is characterized by non-type 2 inflammation, it might explain why we did not find increased Muc5ac expression and in relation no clear alterations in mucus production in this model. In addition, no thickening of the basement membrane or other structural differences were found in this non-allergic model. This is however in line with features observed in non-allergic asthma patients (46–48). Several studies have shown that non-allergic asthma patients have less epithelial damage, less smooth muscle thickening, or other structural changes compared to allergic asthmatic patients (46–49). This suggests that this model resembles some and not all classical features of asthma, but previous studies in human asthma learned that not all these features are to be found in all asthma phenotypes.

As asthmatic patients with underlying neutrophilic inflammation often experience steroid insensitivity (37), we studied the effects of glucocorticosteroids on neutrophilic inflammation and AHR in our model. A slight reduction in AHR combined with a clear reduction in neutrophils in BAL fluid was observed in mice treated with fluticasone propionate. These results show that our model is in fact steroid sensitive, and this is not completely in line with the described literature in asthma patients with neutrophilic inflammation (37). Of note, the concentration of FP (0.05 mg/kg) used in this experiment is comparable to the concentration used in humans, so the question arises whether the steroid insensitivity is due to the neutrophilic airway inflammation or if the inflammation is just a bystander effect. As neutrophils do not play a major role in the induction of airway hyperreactivity in our murine model, we can hypothesize that other mechanisms might be affected by the FP resulting in decreased AHR and inflammation. Additionally, while the dose of FP was comparable to the concentration used in asthma patients, the application times are different. The long-term use of corticosteroids might induce corticosteroid insensitivity of neutrophils. We have to consider that also other corticosteroids than FP might have other effects in our murine model as FP has shown to be prolonging human neutrophil survival (50). Further investigation on a molecular level into the neutrophils in this model might give us more information on why these cells are corticosteroid sensitive.

As ILCs are associated with several features of asthma (11), we investigated changes in the different ILC subtypes after LPS exposure. In absolute numbers, ILC1, NCR^-^ ILC3, and NCR^+^ ILC3 were increased in LPS-instilled mice, whereas no differences in ILC2 numbers were induced by LPS exposure. ILC3 are known to be activated by IL-1β and secrete both IL-17A and IL-22 (34), suggesting that both subtypes of ILC3 could contribute to the increased levels of IL-17A and IL-22 in the airways of LPS-instilled mice.

To investigate the causal role of ILC in the induction of AHR, we exposed SCID mice and Rag2^-/-^γC^-/-^ mice on a BALB/c background to LPS. No AHR was observed in LPS-instilled Rag2^-/-^γC^-/-^ mice. However, LPS-instilled SCID mice had AHR and increased ILC3 proportions in the lung. Additionally, transferring total ILC into Rag2^-/-^γC^-/-^ mice followed by instillation with LPS also re-induced AHR. These results prove that ILCs are important in the induction of AHR after LPS instillation. After adoptive transfer of the total ILC population, ILC2 was the dominant ILC population present in the lungs. This ILC2 dominance is probably due to the plasticity between ILC subtypes and/or mediators that might be missing in the lung environment of Rag2^-/-^γC^-/-^ mice. Our observations about ILC and the AHR are in line with a study in non-neutrophilic and neutrophilic asthmatic patients where IL-22-producing cells were increased in the bronchial lamina propria (51). In addition, in a murine model of non-allergic eosinophilic asthma, ILC2 and ILC3 were activated and induced AHR, suggesting a role for ILCs in induction of AHR in non-allergic asthma models (52). In asthmatic patients with severe asthma, there are some indications pointing toward elevated levels of IL-17 producing ILC3 in BAL fluid (38). Both cytokines seem to be important in the induction of AHR as recently described by Lamb et al. (53). They showed that IL-17A and IL-22 together (by application intratracheally of these cytokines together or separate) but not alone lead to the induction of AHR in BALB/c mice compared to control mice (53), which is in line with our results. Data showing that IL-17A might be involved in AHR in asthmatic patients are, however, rather scarce. Firstly, IL-17A has been implied in enhancing contractile responses of human airway smooth muscle cells in response to methacholine and thus inducing AHR (54). Secondly, Barczyk et al. showed that sputum IL-17A levels correlated negatively with the PC20 (provocative concentration of methacholine causing a 20% fall in FEV_1_), suggesting a role for IL-17A in AHR in asthma (55). Thirdly, other studies have shown links between IL-17A and disease severity (29, 30) which leads us to believe that IL-17A plays a role, though not crucial, in the pathogenesis of asthma.

IL-13, which can be secreted by ILC2, however, is clearly linked to AHR (56). Lung IL-13 levels were still increased in IL-17A^-/-^ mice exposed to LPS which might explain why AHR persists in these mice. Other cytokines such as TNF-⍺ might also be relevant for AHR (57). Neutrophils were still present after depletion of IL-17A which can be explained by high levels of KC and MIP-2 attracting neutrophils to the airways from the bloodstream. In fact, the neutrophilic inflammation does not contribute to the induction of AHR in our model. We depleted neutrophils in the lung *via* anti-Ly6G mAb and analysed AHR after 4 instillations of LPS. No reduction in AHR was noticed in the absence of lung neutrophils. This supports the hypothesis that inflammation on the one hand and AHR on the other hand are two separate mechanisms, each contributing in their own way to the symptoms of asthma (58).

As IL-1β is increased in lungs of LPS-instilled mice, we evaluated the effects of IL-1β blockade on the induction of AHR, blocking the function of IL-1β by blocking the IL-1 receptor with anakinra partially, but significantly reduced AHR in LPS-instilled mice, emphasising that IL-1β and ILC3 might be important in the induction of AHR in this model. Anakinra will block the IL-1 receptor preventing IL-1β from binding and activating the downstream signalling effects, such as upregulation of neutrophil-attracting chemokines (59) leading to a few neutrophils in the BAL fluid of the mice. Secondly, anakinra might also lead to blocking of the autocrine signalling pathway of Il-1β and thus reduced production of IL-1β. This was shown in a model for acute lung injury where IL-1β further upregulated lung inflammation *via* the IL-1β–IL-1 receptor signalling loop (60). These results are in line with a recent study of Kim et al. in obesity-induced asthma where the NLRP3 inflammasome was shown to play a role in the induction of AHR (38). In a proof-of-concept study with healthy volunteers exposed to LPS, anakinra similarly reduced the number of neutrophils and reduced the IL-1β, IL-8, and IL-6 levels in sputum (61). The NLRP3 inflammasome is upregulated in patients with neutrophilic asthma (62). In severe asthmatic patients, NLRP3 and IL-1β mRNA levels in sputum correlated with increased neutrophils and decline in lung function (63). *In vitro* experiments with a bronchial cell line exposed to LPS showed that the respiratory epithelium could be an important source of IL-1β. The mechanism behind IL-1β production is a two-step approach first activating the TLR leading to induction of the NF-κB pathway and production of pro-IL-1β and afterward the cleavage and production of active IL-1β *via* the NLRP3 inflammasome after a second encounter with LPS or other damage-associated molecular patterns (64). This second step might not be present in the *in vitro* culture system explaining the only slight increase of IL-1β protein levels. The LPS–TLR4–epithelium pathway has already been described in a murine model for allergic asthma with HDM (65), making TLR4 and its signalling pathways another target for the next experiments and possible treatment options.

To conclude, in this study we report a murine model in which the external trigger LPS induces airway hyperreactivity and neutrophilic inflammation, thus mimicking certain features of non-allergic neutrophilic asthma. Furthermore, we show that ILC, but not IL-17 or neutrophils, plays a causal role in the induction of AHR in this model. The epithelium–IL-1β–ILC axis might partly be involved in inducing AHR in this murine model, but other currently unrevealed factors should still be identified.

## Data Availability Statement

The raw data supporting the conclusions of this article will be made available by the authors, upon request.

## Ethics Statement

The animal study was reviewed and approved by the Ethical Committee for Animal Research KU Leuven.

## Author Contributions

A-CJ, SS, DB, and J-LC designed the study. A-CJ, BS, and TD conducted the experiments. A-CJ, BS, SS, RS, CB, J-LC, JV, KD, GV, LD, and DB contributed to the data analysis and interpretation of the data and statistics. JC, JV, ED, KD, and GV provided technical support for the experiments and proofread the manuscript. DS provided the SCID mice, and YI provided the IL-17A^-/-^ mice. A-CJ drafted the manuscript, which was critically revised and edited by JV, BS, J-LC, and DB. All authors contributed to the article and approved the submitted version.

## Funding

DB and RS (1805518N) are recipients of a senior researcher fellowship from the Fund for Scientific Research Flanders (FWO). A-CJ is supported by an FWO-SB fellowship (1S20420N). BS is supported by a postdoctoral fellowship of the FWO, Flanders, Belgium (12U6721N). KD was supported by an FWO-SB fellowship (1S77319N). Images were recorded on a Zeiss LSM 780 – SP Mai Tai HP DS (Cell and Tissue Imaging Cluster (CIC)), supported by Hercules AKUL/11/37 and FWO G.0929.15 to Pieter Vanden Berghe, University of Leuven.

## Conflict of Interest

The authors declare that the research was conducted in the absence of any commercial or financial relationships that could be construed as a potential conflict of interest.

## Publisher’s Note

All claims expressed in this article are solely those of the authors and do not necessarily represent those of their affiliated organizations, or those of the publisher, the editors and the reviewers. Any product that may be evaluated in this article, or claim that may be made by its manufacturer, is not guaranteed or endorsed by the publisher.
