# Corrigendum: Innate lymphoid cells are required to induce airway hyperreactivity in a murine neutrophilic asthma model

**DOI:** 10.3389/fimmu.2022.1032423

**Published:** 2022-09-21

**Authors:** Anne-Charlotte Jonckheere, Sven F. Seys, Brecht Steelant, Tatjana Decaesteker, Kaat Dekoster, Jonathan Cremer, Ellen Dilissen, Dominique Schols, Yoichiro Iwakura, Greetje Vande Velde, Christine Breynaert, Rik Schrijvers, Jeroen Vanoirbeek, Jan L. Ceuppens, Lieven J. Dupont, Dominique M. A. Bullens

**Affiliations:** ^1^ Department of Microbiology, Immunology and Transplantation, Allergy and Clinical Immunology Research Group, KU Leuven, Leuven, Belgium; ^2^ Department of Chronic Diseases, Metabolism and Ageing, Laboratory of Respiratory Diseases and Thoracic Surgery, KU Leuven, Leuven, Belgium; ^3^ Department of Imaging and Pathology, Biomedical MRI Unit/Molecular Small Animal Imaging Center (MoSAIC), KU Leuven, Leuven, Belgium; ^4^ Department of Microbiology, Immunology and Transplantation, Laboratory of Virology and Chemotherapy, KU Leuven, Leuven, Belgium; ^5^ Centre for Animal Disease Models, Research Institute for Biomedical Sciences, Tokyo University of Science, Chiba, Japan; ^6^ Department of Public Health and Primary Care, Centre for Environment and Health, KU Leuven, Leuven, Belgium; ^7^ Clinical Division of Respiratory Medicine, UZ Leuven, Leuven, Belgium; ^8^ Clinical Division of Paediatrics, UZ Leuven, Leuven, Belgium

**Keywords:** innate lymphoid cells (ILCs), non-allergic asthma, murine model, neutrophilic inflammation, airway hyperreactivity

In the published article, there was an error made in the provided concentration lipopolysaccharide (LPS) applied endonasally in the murine model for neutrophilic asthma.

A correction has been made to Material and Methods section, “*Neutrophilic asthma model”*. The sentence previously stated:

“Wild-type, SCID, Rag2^-/-^ γC^-/-^, and IL-17A^-/-^ mice were endonasally challenged with 2 μg LPS (in a solution of 50 μl; 40 mg/ml) or 50 μl of saline (0.9% NaCl, B. Braun) on four consecutive days (Supplementary Figure 1A).”

The corrected sentence appears below:

“Wild-type, SCID, Rag2^-/-^ γC^-/-^, and IL-17A^-/-^ mice were endonasally challenged with 2 μg LPS (in a solution of 50 μl; 40 μg/ml) or 50 μl of saline (0.9% NaCl, B. Braun) on four consecutive days (Supplementary Figure 1A).”

The authors apologize for this error and state that this does not change the scientific conclusions of the article in any way. The original article has been updated.

## Publisher’s note

All claims expressed in this article are solely those of the authors and do not necessarily represent those of their affiliated organizations, or those of the publisher, the editors and the reviewers. Any product that may be evaluated in this article, or claim that may be made by its manufacturer, is not guaranteed or endorsed by the publisher.

