# Supplementary material for: Innate Lymphoid Cells Are Required to Induce Airway Hyperreactivity in a Murine Neutrophilic Asthma Model
Source: Front Immunol. 2022 Mar 15;13:849155. doi: 10.3389/fimmu.2022.849155 (PMC8965562; doi:10.3389/fimmu.2022.849155)
Supplement: Supplementary file 1 [file DataSheet_1.docx]

Supplementary Material

# Supplementary Figures

**Supplementary Figure 1: Schematic representations of the murine models and treatment approaches used in the manuscript. A)** Neutrophilic asthma model: endonasal application of LPS on four consecutive days with lung function tests, BAL, lung resection on day 5 **B)** Neutrophil depletion in LPS model with anti-Ly6G (rat IgG2 isotype in controls). **C)** Blocking IL-1 receptor with Anakinra (saline in controls) in LPS model. **D)** Glucocorticoid treatment with fluticasone propionate (FP) or sham (saline + 10% DMSO) in LPS model. All experiments are run in mice with BALB/c background. e.n. = endonasal. i.p. = intraperitoneal.

**Supplementary Figure 2: Gating strategy for neutrophils and ILC on cell suspension isolated from lung tissue.** Neutrophils were gated as Live, CD45^+^, Ly6G^+^ cells. Total ILC population was gated as Live, CD45^+^, Ly6G^-^, Lineage^-^ (CD11b, CD19, CD3e, CD45RB, CD49b, CD5, CD94, TCRγδ and Ter-119), CD90.2^+^, CD127^+^ cells. ILC1 were defined as Live, CD45^+^, Ly6G^-^, Lineage^-^, CD90.2^+^, CD127^+^, KLRG-1^-^, RORγT^-^, NKp46^+^ cells. ILC2 were characterized as Live, CD45^+^, Ly6G^-^, Lineage^-^, CD90.2^+^, CD127^+^, KLRG-1^+^ cells. NCR^-^ ILC3 were gated as Live, CD45^+^, Ly6G^-^, Lineage^-^, CD90.2^+^, CD127^+^, KLRG-1^-^, RORγT^+^, NKp46^-^ cells and NCR^+^ ILC3 as Live, CD45^+^, Ly6G^-^, Lineage^-^, CD90.2^+^, CD127^+^, KLRG-1^-^, RORγT^+^, NKp46^+^ cells. Data are representative for a wild type control mouse. SSC: side scatter. FSC: forward scatter.

**Supplementary Figure 3: Gating strategy for DC on cell suspensions isolated from lung tissue.** Dendritic cells (DC) were gated as low-autofluorescent, CD11c^+^ and MHCII^+^. CD11c^+^ and high-autofluorescent cells were marked as macrophages. Plasmacytoid dendritic cells (pDC) were gated as CD11b^-^ and SiglecH^+^ cells. Conventional dendritic cells (cDC) were subdivided into CD11b^+^CD103^-^ cells and CD11b^-^CD103^+^ cells. Monocyte-derived dendritic cells (moDC) were identified as CD11b^+^CD103^-^CD64^+^. Data are representative for a wild type control mouse. FCS: forward scatter. SSC: side scatter.

**Supplementary Figure 4: Airway resistance, %FEV_0.1_ and BAL neutrophils after different doses of LPS or after different number of applications in BALB/c mice. (A - C)** BALB/c mice were exposed via the nose to LPS 1µg or 2 µg on three (3x) or four (4x) consecutive days. Twenty-four hours after the last LPS challenge, lung function parameters were measured using the FlexiVent. BAL fluid was obtained to determine the differential cell count. Airway resistance (Rn, A) before and after methacholine provocation (0 - 20 mg/ml) were measured in saline-treated BALB/c mice (n = 5), LPS 1 µg-treated BALB/c mice (n = 3), LPS 2 µg (3x)-treated BALB/c mice (n = 3), LPS 2 µg (4x)-treated BALB/c mice (n = 3). Data are represented as mean. Neutrophils (C) were identified in BAL fluid of the same mice and represented as individual values with mean. **(D – F)** BABL/c mice were exposed to 2 µg LPS or saline (50 µl) via the nose on four consecutive days. Twenty-four hours after every application of LPS, airway resistance and %FEV_0.1_ in response to methacholine was measured using the FlexiVent and BAL fluid was obtained to determine differential cell count. Airway resistance (A) and %FEV_0.1_ in response to methacholine (0 – 20 mg/ml) were measured in the different groups. N = 3 in each group and data are represented as mean. Neutrophils (F) in BAL fluid are represented as individual values with mean. *P < 0.05, **P < 0.01; ****P < 0.0001 (saline versus LPS 1µg 4x or LPS 2µg 3x or LPS 2µg 4x).

**Supplementary Figure 5: Mucus production after LPS exposure.** BALB/c wild type (WT) mice were challenged with LPS (2 µg in 50 µl saline) via the nose on four consecutive days (Supplementary Figure 1A). Twenty-four hours after the last challenge with LPS, lung tissue was collected from saline-treated mice (n = 7) and LPS-treated mice (n = 7) for RNA isolation and histology (paraffin sections). Lung Muc5ac (A) mRNA expression was measured by RT-qPCR and results are expressed relative to reference genes β-actin and β-2-microglobulin. A representative image of the Alcian blue and Periodic-acid-Schiff (PAS) staining on paraffin coupes of lung tissue is shown in both saline-treated (B) and LPS-treated (C) mice (40x magnification). Data are presented as individual values with mean and standard deviation. Unpaired t-test was used to determine P-value. **P < 0.01.

**Supplementary Figure 6: Tight junction mRNA expression in respiratory epithelium after LPS exposure.** BALB/c wild type (WT) mice were challenged with LPS (2 µg in 50 µl saline) via the nose on four consecutive days (Supplementary Figure 1A). Twenty-four hours after the last challenge with LPS, lung tissue was collected from saline-treated mice (n = 7) and LPS-treated mice (n = 7) for RNA isolation and histology (paraffin sections). Lung occludin (A), zonula occludens 1 (ZO-1, B), claudin-3 (C), claudin-4 (D) and claudin-18 (E) mRNA expression was measured by RT-qPCR and results are expressed relative to reference genes β-actin and β-2-microglobulin. A representative image of the immunofluorescent staining for occludin, claudin 4 and claudin 3 on paraffin coupes of lung tissue is shown in (F) both in saline-treated and LPS-treated mice. Data are presented as individual values with mean and standard deviation. Unpaired t-test was used to determine P-value. *P < 0.05, **P < 0.01, ***P < 0.001, ****P < 0.0001.

**Supplementary Figure 7: Effect of glucocorticoid treatment on lung ILC proportions in LPS exposed mice.** BALB/c mice were exposed to LPS (2 µg in 50 µl saline) and fluticasone propionate (FP; 0.05mg/kg in 50 µl) via the nose as shown in Supplementary Figure 1D. Forty-eight hours after the last challenge with LPS, lung tissue was resected, and a cell suspension was made for ILC analysis via flow cytometry. (A) Lung ILC1 (CD45^+^ Lin^-^ CD90.2^+^ CD127^+^ KLRG1^-^ ROR𝛾T^-^ NKp46^+^). (B) ILC2 (CD45^+^ Lin^-^ CD90.2^+^ CD127^+^ KLRG1^+^ ROR𝛾T^-^ NKp46^-^). (C) ILC3 (CD45^+^ Lin^-^ CD90.2^+^ CD127^+^ KLRG1^-^ ROR𝛾T^+^) in saline + sham-treated mice (n = 8), saline + FP-treated mice (n = 8), LPS + sham-treated mice (n = 8) and LPS + FP-treated mice (n = 8). Results are expressed as proportions of the total lung ILC population and data are represented as individual values with median and interquartile range. Two independent experiments were combined. Kruskall-Wallis with Dunn’s post hoc test was used to calculate the P-values. *P < 0.05 saline + sham versus LPS + sham). ^$$^P < 0.01 (saline + FP versus LPS + FP).

**Supplementary Figure 8: Lung ILC proportions in wild type BALB/c mice and IL-17A^-/-^** **mice endonasally exposed to saline or LPS.** IL- IL-17A^-/-^ mice (on BALB/c background) were exposed to LPS (2 µg in 50 µl saline) via the nose for four consecutive days (Supplementary Figure 1A). Twenty-four hours after the last challenge with LPS, lung tissue was obtained to isolate cells for flow cytometry identifying the different innate lymphoid cell (ILC) subsets. ILC1 (A; CD45^+^ Lin^-^ CD90.2^+^ CD127^+^ KLRG1^-^ ROR𝛾T^-^ NKp46^+)^, ILC2 (B, CD45^+^ Lin^-^ CD90.2^+^ CD127^+^ KLRG1^+^ ROR𝛾T^-^ NKp46^-^), NCR- ILC3 (C, CD45^+^ Lin^-^ CD90.2^+^ CD127^+^ KLRG1^-^ ROR𝛾T^+^ NKp46^-^) and NCR+ ILC3 (D, CD45^+^ Lin^-^ CD90.2^+^ CD127^+^ KLRG1^-^ ROR𝛾T^+^ NKp46^+^) were characterized in saline-treated wild type mice (n = 6), LPS-treated wild type mice (n = 7), saline-treated IL-17A^-/-^ mice (n = 8) and LPS-treated IL-17A^-/-^ mice (n = 8) and shown as proportions of the total lung ILC population. Data are represented as individual values with median and interquartile range. One-way ANOVA with Sidak post hoc test was used to calculate P-value. ^*^P < 0.05 (WT saline versus WT LPS); ^##^P-value < 0.01 (IL-17^-/-^ saline versus IL-17^-/-^ LPS).

** Supplementary Figure 9: Lung ILC proportions in BALB/c mice endonasally exposed to saline or LPS and treated with anti-Ly6G mAb or isotype control.** Neutrophils were depleted by injecting anti-Ly6G mAb (250 μg) intraperitoneally on day -1, 1 and 3 as shown in Supplementary Figure 1B. Twenty-four hours after the last LPS challenge, lung tissue was obtained to isolate cells and to identify the different innate lymphoid cell (ILC) subsets by flow cytometry. ILC1 (A; CD45^+^ Lin^-^ CD90.2^+^ CD127^+^ KLRG1^-^ ROR𝛾T^-^ NKp46^+)^, ILC2 (B, CD45^+^ Lin^-^ CD90.2^+^ CD127^+^ KLRG1^+^ ROR𝛾T^-^ NKp46^-^), NCR- ILC3 (C, CD45^+^ Lin^-^ CD90.2^+^ CD127^+^ KLRG1^-^ ROR𝛾T^+^ NKp46^-^) and NCR+ ILC3 (D, CD45^+^ Lin^-^ CD90.2^+^ CD127^+^ KLRG1^-^ ROR𝛾T^+^ NKp46^+^) were characterized in saline-treated isotype injected wild type mice (n = 5), LPS-treated isotype injected wild type mice (n = 5) and LPS-treated and anti-Ly6G injected wild type mice (n = 8). Results are shown as proportions of the total lung ILC population. Data are represented as individual values with median and interquartile range. Kruskall-Wallis with Dunn’s post hoc test was used to calculate P-value. ^*^P < 0.05.

**Supplementary Figure 10: Lung ILC proportions in SCID mice exposed to LPS.** SCID mice were endonasally challenged with LPS (2 µg in 50 µl saline) on four consecutive days. Twenty-hours after the last challenge with LPS, lung tissue was obtained to isolate cells and to identify the different innate lymphoid cell (ILC) subsets by flow cytometry. ILC1 (A; CD45^+^ Lin^-^ CD90.2^+^ CD127^+^ KLRG1^-^ ROR𝛾T^-^ NKp46^+)^, ILC2 (B, CD45^+^ Lin^-^ CD90.2^+^ CD127^+^ KLRG1^+^ ROR𝛾T^-^ NKp46^-^), NCR- ILC3 (C, CD45^+^ Lin^-^ CD90.2^+^ CD127^+^ KLRG1^-^ ROR𝛾T^+^ NKp46^-^) and NCR+ ILC3 (D, CD45^+^ Lin^-^ CD90.2^+^ CD127^+^ KLRG1^-^ ROR𝛾T^+^ NKp46^+^) were characterized in saline-treated SCID mice (n = 10) and LPS-treated SCID mice (n = 10). Results are shown as proportions of the total lung ILC population. All data are represented as individual values with median and interquartile range. Unpaired t-test was used to calculate P-value. ^****^P-value < 0.0001.

**Supplementary Figure 11: Lung ILC proportions in SCID mice exposed to LPS and treated with anakinra.** SCID mice were endonasally treated with anakinra (50 μl of a 20 mg/ml solution) on day -1, 1 and 3 together with the four endonasal challenges with LPS (2 µg in 50 µl saline) as shown in Supplementary Figure 1C. Twenty-four hours after the last challenge with LPS, lung tissue was obtained to identify the different ILC subsets. ILC1 (A; CD45^+^ Lin^-^ CD90.2^+^ CD127^+^ KLRG1^-^ ROR𝛾T^-^ NKp46^+)^, ILC2 (B, CD45^+^ Lin^-^ CD90.2^+^ CD127^+^ KLRG1^+^ ROR𝛾T^-^ NKp46^-^), ILC3 (C, CD45^+^ Lin^-^ CD90.2^+^ CD127^+^ KLRG1^-^ ROR𝛾T^+^) were characterized in saline + sham- (n = 7), saline + anakinra- (n = 5), LPS + sham- (n = 7) and LPS + anakinra-treated SCID mice (n = 9). Results are shown as % of total ILC population. All data are represented as individual values with median and interquartile range.

**Supplementary Figure 12: *In vitro* stimulation of Calu-3 epithelial cells with LPS.** Calu-3 epithelial cells were stimulated with LPS (1 µg/ml) for 6 hours or 48 hours. Control cells were kept in medium alone. After 6 hours of stimulation, Calu-3 cells were collected and total RNA was extracted. mRNA expression of IL-1β (A), IL-6 (B), IL-8 (C) and IL-33 (D) are shown relative to reference genes β-actin and guanine nucleotide-binding protein subunit beta-2-like 1 (GNB2L1). After 48 hours of culture, supernatants were collected and IL-1β (E), IL-6 (F), IL-8 (G) and TNF-α (H) levels were measured with ELISA. Data are represented as bars with mean and standard deviation. Each bar graph contains duplicates of the same experiment.

**Supplementary Figure 13: Proportion of ILC among CD45+ cells in the lungs of wild type, SCID and Rag2^-/-^ γC^-/-^** **mice exposed to saline or LPS.** Wild type (n = 7), SCID (n= 10), Rag2^-/-^ γC^-/-^ (n = 6) mice were endonasally exposed to LPS (2 µg in 50 µl saline) on four consecutive days (Supplementary Figure 1A). Twenty-four hours after the last challenge with LPS, lung tissue was obtained to isolate cells and to identify the total ILC population by flow cytometry. Total ILC (CD45^+^ Lin^-^ CD90.2^+^ CD127^+^) is shown as % of CD45+ cells. All data are represented as individual values with mean.

# Supplementary Tables

**Supplementary Table 1:** primer and probe sequences used for qPCR on mouse lung tissue

| β-actin | FW | aga ggg aaa tcg tgc gtg ac |
| --- | --- | --- |
|  | RV | caa tag tga tga cct ggc cgt |
|  | TP | cac tgc cgc atc ctc ttc ctc cc |
| Cldn1 | FW | ccc atc aat gcc agg tat gaa |
|  | RV | ggt aag agg ttg ttt tcc ggg |
|  | TP | ctt tac tgg ctg ggc tgc tgc c |
| Cldn3 | FW | act acc ggg cct agg aac tgt c |
|  | RV | caa gta gct gca gtg gcc ac |
|  | TP | aag ccg aat gga caa aga aac ctc gc |
| Cldn4 | FW | cga gca cag ctg gtc cta cc |
|  | RV | aag ggt tcc atg gca gag c |
|  | TP | cat ggt gtg ctg agt gac tga ctg agg g |
| Cldn18 | FW | atc atc tcc ggc atc tgt gc |
|  | RV | ggt gta cct ggt ctg aac ggt c |
|  | TP | ttt gcc aac atg ctg gtg acc aac tt |
| Ocln | FW | aca aga gaa att ttg atg cag gtc t |
|  | RV | cat cag cag cag cca tgt act c |
|  | TP | aag agc tta cag gca gaa cta gac gac gtc aa |
| B2M | FW | ctg aag ctg aca gca ttc gg |
|  | RV | ctt tgg agt acg ctg gat agc c |
|  | TP | aga tgt ctc gct ccg tgg cct tag c |
| Tjp1 (=ZO-1) | FW | ttc gag aag ctg gat tcc taa gac |
|  | RV | cag tcc cag cat ctc gtg g |
|  | TP | cat ctt tgg acc aat agc tga tgt tgc ca |
| Muc5ac | FW | caa tgc aca ggc cag gct |
|  | RV | ggg tac caa cat gcc ctt ga |
|  | TP | act ctc tga aat cgt acc atg aac acc gct |

FW = forward primer, RV = reverse primer, TP = taqman probe. Cldn = claudin, Ocln = occludin, ZO-1 = zonula occludens 1 and B2M = β_2_-microglobulin.

**Supplementary Table 2**: fluorochrome-conjugated anti-mouse antibodies and specifications used for flow cytometry

| **Marker** | **Fluorochrome** | **Clone** | **Supplier** | **Catalog number** |
| --- | --- | --- | --- | --- |
| CD11b | FITC | M1/70 | BD | 557396 |
| CD19 | FITC | 1D3 | BD | 557398 |
| CD3e | FITC | 145 – 2C11 | BD | 553062 |
| CD45RB | FITC | 16A | BD | 553100 |
| CD49b | FITC | DX5 | BD | 553857 |
| CD5 | FITC | 53 – 7.3 | BD | 553021 |
| CD94 | FITC | 18d3 | Biolegend | 105506 |
| TCR gd | FITC | GL3 | BD | 553177 |
| Ter-119 | FITC | TER - 119 | BD | 557915 |
| Ly-6G | Pe-Dazzle 594 | 1A8 | Biolegend | 127648 |
| CD45 | AF-700 | 30 – F11 | BD | 560510 |
| CD90.2 | PE-Cy7 | 53 – 2.1 | BD | 561642 |
| CD127 | PE | SB/199 | BD | 552543 |
| KLRG-1 | BV421 | 2F1 | BD | 562897 |
| NKp46 | BV711 | 29A1.4 | Biolegend | 137621 |
| RORyT | APC | AFKJS-9 | Thermo Fisher | 17-6988-82 |
| CD11c | Pe-Dazzle 594 | N418 | Biolegend | 117348 |
| CD45 | BV711 | 30 – F11 | Biolegend | 103147 |
| CD11b | BV605 | M1/70 | Biolegend | 101257 |
| MHCII | APC-Cy7 | M5/144.15.2 | Biolegend | 107268 |
| CD103 | BV421 | 2E7 | Biolegend | 121422 |
| CD64 | APC | X54-5/7.1 | Biolegend | 139306 |
| Siglec H | PE | 551 | Biolegend | 129606 |

**Supplementary Table 3:** Configuration of LSR Fortessa SORP flow cytometer (BD biosciences).

| Laser Wavelength (nm) | Laser Power (mW) | Detector | Spectral Range (nm) | Dichroic LP Filter (nm) | Band Pass Filter (nm) | Fluorochrome detected |
| --- | --- | --- | --- | --- | --- | --- |
| 405 (violet) | 50 | V785 | 755 – 815 | 735 | 785/60 | N/A |
|  |  | V711 | 698.5 -723.5 | 685 | 711/25 | Brilliant Violet 711 |
|  |  | V661 | 651-671 | 630 | 661/20 | N/A |
|  |  | V610 | 600-620 | 600 | 610/20 | Brilliant Violet 605 |
|  |  | V525 | 505-535 | 505 | 525/50 | Zombie Aqua |
|  |  | V450 | 425-475 | - | 450/50 | Brilliant Violet 421 |
| 488 (blue) | 50 | B710 | 685-735 | 685 | 710/50 | N/A |
|  |  | B530 | 515-545 | 505 | 530/30 | FITC, autofluorescence |
|  |  | SSC | 483-493 | - | 488/10 | Side scatter detection |
| 561 (yellow-green) | 50 | Y780 | 750-810 | 750 | 780/60 | PE-Cy7 |
|  |  | Y710 | 685-735 | 685 | 710/50 | N/A |
|  |  | Y670 | 655-685 | 635 | 670/30 | N/A |
|  |  | Y610 | 600-620 | 600 | 610/20 | PE-Dazzle594 |
|  |  | Y585 | 578-592 | - | 585/15 | PE |
| 640 (red) | 40 | R780 | 750-810 | 750 | 780/60 | FVD eF780,  APC-Cy7 |
|  |  | R730 | 708-750 | 690 | 730/45 | Alexa Fluor 700 |
|  |  | R670 | 663-677 | - | 670/14 | APC |

**Supplementary Table 4:** primer and probe sequences used for qPCR on Calu-3 cells

| β-actin | FW | gga cat ccg caa aga cct gt |
| --- | --- | --- |
|  | RV | ctc agg agg agc aat gat ctt gat |
|  | TP | ctg gcg gca cca cca tgt acc ct |
| GNB2L1 | FW | cac tgt cca gga tga gag cca |
|  | RV | cat acc ttg acc agc ttg tcc c |
|  | TP | tcc gct tct cgc cca aca gca g |
| IL-1β | FW | ttg ctc aag tgt ctg aag cag c |
|  | RV | caa gtc atc ctc att gcc act g |
|  | TP | tac ctg agc tcg cca gtg aaa tga tgg |
| IL-6 | FW | cca gga gcc cag cta tga ac |
|  | RV | aag gca gca ggc aac acc |
|  | TP | cct tct cca caa gcg cct tcg gt |
| IL-8 | FW | cac tgc gcc aac aca gaa att a |
|  | RV | att ctc agc cct ctt caa aaa ctt c |
|  | TP | agc tct gtc tgg acc cca agg aaa act g |
| IL-33 | FW | atg aat cag gtg acg gtg ttg a |
|  | RV | gcc tgg tct ggc agt ggt t |
|  | TP | cca aca aca agg aac act ctg tgg agc tc |

FW = forward primer, RV = reverse primer, TP = taqman probe. GNB2L1 = guanine nucleotide-binding protein subunit beta-2-like. IL = interleukin

**Supplementary Table 5**: Effect of glucorticosteroids on lung cytokines in LPS-treated mice

|  | **Saline + sham**  **(n = 8)** | **Saline + FP**  **(n = 8)** | **LPS + sham**  **(n=8)** | **LPS + FP**  **(n = 8)** | **P-value** |
| --- | --- | --- | --- | --- | --- |
| **TNF-α** | 0.546  (0.049 – 2.641) | 0.071  (0.036 – 0.112) | 0.494  (0.351 – 0.698) | 0.334  (0.071 – 0.456) | 0.0772 |
| **IL-1β** | 7.529  (0.399 – 34.44) | 0.639  (0.387 – 0.835) | 8.688  (7.0 – 14.32) | 6.585  (1.679 – 8.612) | 0.0491 |
| **MIP-2** | 0.381  (0.275 – 0.517) | 0.499  (0.287 – 1.012) | 2.029  (1.478 – 2.798)^*^ | 1.37  (0.258 – 2.217) | 0.0461 |
| **KC** | 0.81  (0.719 – 1.017) | 0.945  (0.661 – 1.179) | 5.363  (4.108 – 6.475)^*^ | 3.579  (0.985 – 5.378) | 0.0081 |
| **IL-6** | 0  (0 – 4.454) | 0  (0 – 0.465) | 1.37  (0.552 – 6.783) | 0.616  (0.120 – 0.907) | 0.0698 |
| **IL-13** | 0.414  (0 – 0.85) | 0  (0 – 0.278) | 0.349  (0.261 – 0.424) | 0.278  (0 – 0.513) | 0.3993 |
| **IL-17A** | 0.001  (0 – 0.002) | 0.002  (0 – 0.004) | 0.002  (0 – 0.004) | 0.001  (0 – 0.003) | 0.8969 |

Values are expressed as pg per g lung tissue and presented as median with 25 and 75 percentiles between brackets. Kruskall-Wallis with Dunn’s post hoc test was used to calculate the P-value. Murine model is described in Supplementary Figure 1D. ^*^P < 0.05 (saline + sham versus LPS + sham).

**Supplementary Table 6**: Cytokine production in lung tissue of saline-treated and LPS-treated wild type and IL-17A^-/-^ mice.

|  | **Wild type**  **Saline-treated**  **(n = 7)** | **Wild type**  **LPS-treated**  **(n = 7)** | **IL-17A^-/-^**  **Saline-treated**  **(n = 8)** | **IL-17A^-/-^**  **LPS-treated**  **(n = 8)** | **P-value** |
| --- | --- | --- | --- | --- | --- |
| **TNF-α** | 0.07  (0.05 – 0.11) | 6.3  (2.7 – 8.7)^**^ | 0.13  (0.09 – 0.21) | 7.8  (2.4 – 10.1)^##^ | < 0.0001 |
| **IL-1β** | 0.69  (0.52 – 1.19) | 73.9  (58.1 – 140.1)^**^ | 0.79  (0.47 – 1.42) | 74.6  (32.5 – 82.9)^##^ | < 0.0001 |
| **MIP-2** | 0.52  (0.49 – 1.38) | 26.9  (14.2 – 34.7)^*^ | 0.68  (0.38 – 0.91) | 34.9  (17.5 – 48.8)^##^ | < 0.0001 |
| **KC** | 1.12  (1.11 – 1.57) | 22.9  (14 – 33.6)^**^ | 1.23  (0.79 – 2.1) | 28.7  (20.2 – 36.6)^##^ | < 0.0001 |
| **IL-6** | 0.06  (0 – 0.1) | 20.7  (5.6 – 22.5)^**^ | 0.006  (0 – 0.24) | 21.4  (10.5 – 29.9)^##^ | < 0.0001 |
| **IL-13** | 0  (0 – 0.16) | 1.01  (0.72 – 1.27)^**^ | 0.02  (0 – 0.27) | 1.16  (0.66 – 1.61)^##^ | < 0.0001 |
| **IL-17A** | 0.8  (0.5 – 1.2) | 32.2  (15.9 – 93.9)^*^ | 0  (0 – 0.003)^$^ | 0  (0 – 0.001)^££££^ | < 0.0001 |

Values are expressed as pg per mg lung tissue and presented as median with 25 and 75 percentiles between brackets. Kruskal-Wallis with Dunn’s post hoc test was used to calculate the P-value. *P < 0.05, **P < 0.01 (WT saline versus WT LPS); ^##^P < 0.01 (IL-17A^-/-^ saline versus IL-17A^-/-^ LPS); ^$^P < 0.05 (WT saline versus IL-17A^-/-^ saline); ^££££^P < 0.0001 (WT LPS versus IL-17A^-/-^ LPS).

**Supplementary Table 7**: Cytokine production in lung tissue of saline-treated, LPS-treated and LPS + anti-Ly6G-treated mice.

|  | **Saline + isotype**  **(n = 5)** | **LPS + isotype**  **(n = 5)** | **LPS + anti-Ly6G (n = 8)** | **P-value** |
| --- | --- | --- | --- | --- |
| **TNF-α** | 0.45  (0.24 – 0.67) | 4.43  (3.078 – 6.32) | 14.99  (7.19 – 32.2)^***^ | < 0.0001 |
| **IL-1β** | 0.5  (0.42 – 0.89) | 126.3  (82.1 – 178.9) | 379.5  (212.1 – 577.1)^***^ | < 0.0001 |
| **MIP-2** | 1.02  (0.55 – 1.48) | 34.6  (22.1 – 40.9) | 60.9  (48.9 – 150.7)^***^ | < 0.0001 |
| **KC** | 0.79  (0.57 – 0.97) | 10.1  (7.9 – 28.8) | 22.3  (12.02 – 43.9)^**^ | 0.0004 |
| **IL-6** | 0.19  (0 – 0.42) | 17.9  (5.3 – 31.8) | 58.3  (29.4 – 136.4)^***^ | < 0.0001 |
| **IL-13** | 0  (0 – 115.5) | 0  (0 – 1017) | 7.5  (1.09 – 13.5) | 0.0369 |
| **IL-17A** | 5.13  (2.5 – 7.2) | 37.8  (28.5 – 146.5) | 297.1  (198.7 – 652.6)^**^ | 0.0002 |

Values are expressed as pg per mg lung tissue and presented as median with 25 and 75 percentiles between brackets. Kruskal-Wallis test was used to calculate the P-value. ^**^P < 0.01, ^***^P < 0.001 (saline + isotype versus LPS + anti-Ly6G).
